# Supplementary material for: A Scalable Computational Framework for Establishing Long-Term Behavior of Stochastic Reaction Networks
Source: PLoS Comput Biol. 2014 Jun 26;10(6):e1003669. doi: 10.1371/journal.pcbi.1003669 (PMC4072526; doi:10.1371/journal.pcbi.1003669)
Supplement: Text S1 — Text supporting information file with full mathematical proofs and more detailed examples. (PDF) [file pcbi.1003669.s001.pdf]

Supplementary material

S1 Proofs of the main results

Consider a reaction network with d species $\boldsymbol{S_1}, \dots, \boldsymbol{S_d}$ and K reaction channels with stoichiometric vectors $\zeta_1, \dots, \zeta_K$. Let $\mathbb{N}_0$ be the set of non-negative integers. The state of the system at any time is given by a vector $x = (x_1, \dots, x_d) \in \mathbb{N}_0^d$, where x_i is the number of molecules of $\boldsymbol{S_i}$. When the state is x, the j-th reaction fires at rate $\lambda_j(x)$ and changes the state by ζ_j. We can represent the reaction dynamics by a Markov process with state space $\mathcal{S} \subset \mathbb{N}_0^d$, where $\mathcal{S}$ satisfies the following property: if $x \in \mathcal{S}$ and $\lambda_k(x) > 0$ for some $k = 1, \dots, K$, then $x + \zeta_k \in \mathcal{S}$. This ensures that if the reaction dynamics starts in $\mathcal{S}$, then it stays in $\mathcal{S}$ forever.

The rate of change of the distribution of a Markov process is given by its generator, which is an operator that maps functions to functions (see Chapter 4 in Ethier and Kurtz [6]). The generator of the Markov process corresponding to our reaction network is given by

$$\mathbb{A}f(x) = \sum_{k=1}^{K} \lambda_k(x) \Delta_{\zeta_k} f(x), \tag{S1.1}$$

where $f : \mathbb{N}_0^d \to \mathbb{R}$ and for any vector $\zeta \in \mathbb{Z}^d$

$$\Delta_\zeta f(x) = f(x + \zeta) - f(x).$$

Our proofs will depend on an important relation, called Dynkin's formula (see Lemma 19.21 in Kallenberg [9]). Let $(X_{x_0}(t))_{t \geq 0}$ be the Markov process with generator $\mathbb{A}$ and initial state x_0. If $f : \mathbb{N}_0^d \to \mathbb{R}$ is a bounded function, then Dynkin's formula states that

$$\mathbb{E}\left(f(X_{x_0}(\tau))\right) = f(x_0) + \mathbb{E}\left(\int_0^\tau \mathbb{A}f(X_{x_0}(s))ds\right) \tag{S1.2}$$

for any stopping time τ which is finite almost surely. This relation will also hold for a general function f if there exists a finite set $A \subset \mathcal{S}$ such that $X_{x_0}(t) \in A$ for all $t \leq \tau$. In this case we also have

$$\mathbb{E}\left(g(\tau) f(X_{x_0}(\tau))\right) = g(0) f(x_0) + \mathbb{E}\left(\int_0^\tau \left(f(X_{x_0}(s)) \frac{dg(s)}{ds} + g(s) \mathbb{A}f(X_{x_0}(s)) \right) ds \right) \tag{S1.3}$$

for any differentiable function $g : (0, \infty) \to \mathbb{R}$ and any function $f : \mathbb{N}_0^d \to \mathbb{R}$.

We recall the main condition of our paper below.

Condition S1.1. (Drift-Diffusivity Condition) *For a positive vector $v \in \mathbb{R}^d$ there exist positive constants c_1, c_2, c_3, c_4 and a nonnegative constant c_5 such that for all $x \in \mathcal{S}$*

$$\sum_{k=1}^{K} \lambda_k(x) \langle v, \zeta_k \rangle \leq c_1 - c_2 \langle v, x \rangle \tag{S1.4a}$$

$$\text{and} \sum_{k=1}^{K} \lambda_k(x) \langle v, \zeta_k \rangle^2 \leq c_3 + c_4 \langle v, x \rangle + c_5 \langle v, x \rangle^2. \tag{S1.4b}$$

This *drift-diffusivity* condition is called Condition **DD**, from now on. We normalize the vector v in this condition to satisfy

$$\max\{|\langle v, \zeta_k \rangle| : k = 1, \dots, K\} = 1. \tag{S1.5}$$

Corresponding to the positive vector v we define the *v-norm* on $\mathbb{R}^d$ by

$$\|x\|_v = \sum_{i=1}^{d} v_i |x_i|. \tag{S1.6}$$

We now provide some intuition on how Condition **DD** arises and how it is used. Let f be the function defined by $f(x) = \|x\|_v = \langle v, x\rangle$. Then from (S1.1) we obtain

$$\mathbb{A}f(x) = \sum_{k=1}^{K} \lambda_k(x)\langle v, \zeta_k\rangle.$$

For now assume that $\sup_{t\geq 0} \mathbb{E}(\|X_{x_0}(t)\|_v) < \infty$ and $\sup_{t\geq 0} \mathbb{E}\left(\int_0^t |\mathbb{A}f(X_{x_0}(s))|\, ds\right) < \infty$. Since $\mathbb{A}$ is the generator of the Markov process $(X_{x_0}(t))_{t\geq 0}$, it follows that the process $(m(t))_{t\geq 0}$ defined by

$$m(t) = \|X_{x_0}(t)\|_v - \|x_0\|_v - \int_0^t \mathbb{A}f(X_{x_0}(s))ds,$$

is a local martingale (see Chapter 4 in [6]). Hence we can write the stochastic equation for the dynamics of $(\|X_{x_0}(t)\|_v)_{t\geq 0}$ as

$$\|X_{x_0}(t)\|_v = \|x_0\|_v + \int_0^t \mathbb{A}f(X_{x_0}(s))ds + m(t). \tag{S1.7}$$

This equation shows that at time t, the process $(\|X_{x_0}(t)\|_v)_{t\geq 0}$ experiences a *drift* given by

$$\mathbb{A}f(X_{x_0}(t)) = \sum_{k=1}^{K} \lambda_k(X_{x_0}(t))\langle v, \zeta_k\rangle.$$

This drift provides a direction to the dynamics. On the other hand, the martingale term $m(t)$ captures the *diffusive* effects at time t, because it causes *undiectional* perturbations in the dynamics. The *predictable quadratic variation* (see Chapter 26 in [9]) of the martingale $(m(t))_{t\geq 0}$ is

$$\langle m\rangle_t = \int_0^t \sum_{k=1}^{K} \lambda_k(X_{x_0}(s))\langle v, \zeta_k\rangle^2 ds, \tag{S1.8}$$

which shows that at time t, the strength of the diffusive effects in the dynamics of $(\|X_{x_0}(t)\|_v)_{t\geq 0}$ is

$$\sum_{k=1}^{K} \lambda_k(X_{x_0}(t))\langle v, \zeta_k\rangle^2.$$

Therefore Condition **DD** provides upper bounds on the drift and diffusion components in the dynamics of $(\|X_{x_0}(t)\|_v)_{t\geq 0}$ when $X_{x_0}(t) = x$. Hence we call this condition as the *drift-diffusivity* condition. Observe that when the process $(\|X_{x_0}(t)\|_v)_{t\geq 0}$ goes above c_1/c_2 then it experiences a negative drift, indicating that it will move downwards. This fact is crucial for proving our results.

S1.1 Moment Bounds

Let $(X_{x_0}(t))_{t\geq 0}$ be the Markov process with generator $\mathbb{A}$ and initial state $x_0 \in \mathcal{S}$. Its distribution at time t is denoted by $p_{x_0}(t)$. Hence for any $y \in \mathcal{S}$

$$p_{x_0}(t, y) = \mathbb{E}(X_{x_0}(t) = y).$$

From now on we suppose that Condition **DD** is satisfied for some positive vector v scaled according to (S1.5). For any positive integer r define

$$m^r_{x_0}(t) = \mathbb{E}\left(\|X_{x_0}(t)\|^r_v\right) = \sum_{x\in\mathcal{S}} \|x\|^r_v p_{x_0}(t, x). \tag{S1.9}$$

For $j = 0, 1, \ldots, (r-1)$ let

$$\kappa_j^r = \begin{cases} \binom{r}{r-j} c_3 + \binom{r}{r-j+1} c_4 + \binom{r}{r-j+2} c_5 & \text{for } j = 0, \ldots, r-2 \\ r c_1 + \binom{r}{2} c_4 + \binom{r}{3} c_5 & \text{for } j = r-1, \end{cases}$$

where $\binom{i}{j} = \frac{i!}{j!(i-j)!}$ if $i \geq j$ and 0 otherwise. Define

$$\beta_r = r\left(c_2 - \left(\frac{r-1}{2}\right) c_5\right) \tag{S1.10}$$

and let

$$r_{\max} = \begin{cases} 1 + \frac{2c_2}{c_5} & \text{if } c_5 > 0 \\ \infty & \text{if } c_5 = 0. \end{cases} \tag{S1.11}$$

Note that for any positive integer $r < r_{\max}$ we have $\beta_r > 0$. Define

$$C_r(x_0) = \max\left\{ \|x_0\|_v^r, \frac{1}{\beta_r}\sum_{j=0}^{r-1} \kappa_j^r C_j(x_0) \right\} \quad \text{and} \quad \hat{C}_r = \frac{1}{\beta_r}\sum_{j=0}^{r-1} \kappa_j^r \hat{C}_j,$$

where $C_0(x_0) = \hat{C}_0 = 1$. Let $U_{x_0}^0(t) = 1$ for all $t \geq 0$. For any integer $r \geq 1$ and $t \geq 0$ define

$$U_{x_0}^r(t) = e^{-\beta_r t}\|x_0\|_v^r + \sum_{j=0}^{r-1} \kappa_j^r \int_0^t e^{-\beta_r (t-s)} U_{x_0}^j(s) ds.$$

We now present a result that is slightly more general than Theorem 2 in the paper.

Theorem S1.2. *Assume that Condition* **DD** *holds. For any positive integer r and $x_0 \in \mathcal{S}$ let $m_{x_0}^r(t)$ be given by* (S1.9)*. If $r < r_{\max}$ then we have the following.*

(A) For any $t \geq 0$, $m_{x_0}^r(t) \leq U_{x_0}^r(t)$.

(B) For any $x_0 \in \mathcal{S}$, $\sup_{t \geq 0} m_{x_0}^r(t) \leq C_r(x_0)$.

(C) For all $x_0 \in \mathcal{S}$, $\limsup_{t\to\infty} m_{x_0}^r(t) \leq \hat{C}_r$.

Proof. Let $f(x) = \langle v, x\rangle^r$ for some integer $r \geq 1$. Then

$$\begin{aligned} \mathbb{A} f(x) &= \sum_{k=1}^K \lambda_k(x)\left((\langle v, x\rangle + \langle v, \zeta_k\rangle)^r - \langle v, x\rangle^r\right) \\ &= \sum_{i=1}^r \binom{r}{i}\left(\sum_{k=1}^K \lambda_k(x)\langle v, \zeta_k\rangle^i\right)\langle v, x\rangle^{r-i} \\ &= r\left(\sum_{k=1}^K \lambda_k(x)\langle v, \zeta_k\rangle\right)\langle v, x\rangle^{r-1} + \sum_{i=2}^r \binom{r}{i}\left(\sum_{k=1}^K \lambda_k(x)\langle v, \zeta_k\rangle^i\right)\langle v, x\rangle^{r-i}. \end{aligned}$$

Due to (S1.5) and (S1.4b) we get that for $i \geq 2$

$$\left(\sum_{k=1}^K \lambda_k(x)\langle v, \zeta_k\rangle^i\right) \leq \left(\sum_{k=1}^K \lambda_k(x)\langle v, \zeta_k\rangle^2\right) \leq c_3 + c_4\langle v, x\rangle + c_5\langle v, x\rangle^2$$

and hence using (S1.4a) we obtain

$$\begin{aligned}
\mathbb{A}f(x) &\leq c_1 r\langle v,x\rangle^{r-1} - \beta_r\langle v,x\rangle^r + \sum_{i=2}^{r}\binom{r}{i}\langle v,x\rangle^{r-i}\left(c_3 + c_4\langle v,x\rangle + c_5\langle v,x\rangle^2\right)\\
&= \sum_{j=0}^{r-1}\kappa_j^r\langle v,x\rangle^j - \beta_r\langle v,x\rangle^r\\
&= -\beta_r H_r(\langle v,x\rangle),
\end{aligned} \tag{S1.12}$$

where H_r is the polynomial given by

$$H_r(y) = y^r - \frac{1}{\beta_r}\sum_{j=0}^{r-1}\kappa_j^r y^j. \tag{S1.13}$$

Let $g(t) = e^{\beta_r t}$ and $f(x) = \langle v,x\rangle^r$. Then using (S1.12) we get

$$\begin{aligned}
\frac{dg(t)}{dt}f(x) + g(t)\mathbb{A}f(x) &\leq \beta_r e^{\beta_r t}\langle v,x\rangle^r - \beta_r e^{\beta_r t}H_r(\langle v,x\rangle)\\
&= e^{\beta_r t}\sum_{j=0}^{r-1}\kappa_j^r\langle v,x\rangle^j.
\end{aligned}$$

Let $(X_{x_0}(t))_{t\geq 0}$ be the Markov process with generator $\mathbb{A}$ and initial state x_0. Pick a $M > 0$ and let τ_M be the stopping time given by

$$\tau_M = \inf\{t \geq 0 : \langle v, X_{x_0}(t)\rangle \geq M\}.$$

Then (S1.3) implies that

$$\mathbb{E}\left(e^{\beta_r(t\wedge\tau_M)}\langle v, X_{x_0}(t\wedge\tau_M)\rangle^r\right) \leq \langle v,x_0\rangle^r + \mathbb{E}\left(\int_0^{t\wedge\tau_M} e^{\beta_r s}\sum_{j=0}^{r-1}\kappa_j^r\langle v, X_{x_0}(s)\rangle^j ds\right) \tag{S1.14}$$

for any $t \geq 0$. We will prove all parts of this theorem by induction. Part (A) is trivially true for $r = 0$. Assume that it holds for all integers less than $n < (r_{\max} - 1)$. Also suppose that $\tau_M \to \infty$ a.s. as $M \to \infty$. Letting $M \to \infty$ in (S1.14) and using the dominated convergence theorem along with Fatou's lemma yields

$$\begin{aligned}
\mathbb{E}\left(e^{\beta_n t}\langle v, X_{x_0}(t)\rangle^n\right) &= \mathbb{E}\left(\lim_{M\to\infty} e^{\beta_n(t\wedge\tau_M)}\langle v, X_{x_0}(t\wedge\tau_M)\rangle^n\right)\\
&\leq \langle v,x_0\rangle^n + \lim_{M\to\infty}\mathbb{E}\left(\int_0^{t\wedge\tau_M} e^{\beta_n s}\sum_{j=0}^{n-1}\kappa_j^n\langle v, X_{x_0}(s)\rangle^j ds\right)\\
&\leq \langle v,x_0\rangle^n + \sum_{j=0}^{n-1}\kappa_j^n\int_0^t e^{\beta_n s}\mathbb{E}\left(\langle v, X_{x_0}(s)\rangle^j\right) ds\\
&= \langle v,x_0\rangle^n + \sum_{j=0}^{n-1}\kappa_j^n\int_0^t e^{\beta_n s} m_{x_0}^j(s) ds\\
&\leq \langle v,x_0\rangle^n + \sum_{j=0}^{n-1}\kappa_j^n\int_0^t e^{\beta_n s} U_{x_0}^j(s) ds.
\end{aligned}$$

Multiplying both sides by $e^{-\beta_n t}$ gives us

$$m_{x_0}^n(t) = \mathbb{E}\left(\langle v, X_{x_0}(t)\rangle^n\right) \leq U_{x_0}^n(t) = e^{-\beta_n t}\langle v,x_0\rangle^n + \sum_{j=0}^{n-1}\kappa_j^n\int_0^t e^{-\beta_n(t-s)}U_{x_0}^j(s)ds.$$

This proves part (A), provided we can show that $\tau_M \to \infty$ a.s. as $M \to \infty$. For this note that (S1.14) for $r = 1$ yields

$$\begin{aligned}\mathbb{E}\left(e^{c_2(t\wedge\tau_M)}\langle v, X_{x_0}(t\wedge\tau_M)\rangle\right) &\leq \langle v, x_0\rangle + c_1\mathbb{E}\left(\int_0^{t\wedge\tau_M} e^{c_2 s}ds\right)\\ &= \langle v, x_0\rangle + \frac{c_1}{c_2}\mathbb{E}\left(e^{c_2(t\wedge\tau_M)} - 1\right)\\ &\leq \langle v, x_0\rangle + \frac{c_1}{c_2}\left(e^{c_2 t} - 1\right).\end{aligned}$$

Markov's inequality implies that

$$\begin{aligned}\mathbb{P}\left(\tau_M < t\right) &= \mathbb{P}\left(\langle v, X_{x_0}(t\wedge\tau_M)\rangle \geq M\right)\\ &\leq \frac{\mathbb{E}(\langle v, X_{x_0}(t\wedge\tau_M)\rangle)}{M}\\ &\leq \frac{\mathbb{E}\left(e^{c_2(t\wedge\tau_M)}\langle v, X_{x_0}(t\wedge\tau_M)\rangle\right)}{M}\\ &\leq \frac{\langle v, x_0\rangle + \frac{c_1}{c_2}\left(e^{c_2 t} - 1\right)}{M}.\end{aligned}$$

Hence for any $t \geq 0$

$$\lim_{M\to\infty}\mathbb{P}\left(\tau_M < t\right) = 0.$$

This shows that $\tau_M \to \infty$ a.s. as $M \to \infty$ and completes the proof of part (A).

The following inequality certainly holds for $r = 0$.

$$\sup_{t\geq 0} U^r_{x_0}(t) \leq C_r(x_0).$$

Assume that it holds for all integers r less than $n < (r_{\max} - 1)$. Then

$$\begin{aligned}U^n_{x_0}(t) &= e^{-\beta_n t}\langle v, x_0\rangle^n + \sum_{j=0}^{n-1}\kappa^n_j\int_0^t e^{-\beta_n(t-s)}U^j_{x_0}(s)ds\\ &\leq e^{-\beta_n t}\langle v, x_0\rangle^n + \sum_{j=0}^{n-1}\kappa^n_j C_j(x_0)\int_0^t e^{-\beta_n(t-s)}ds\\ &= e^{-\beta_n t}\langle v, x_0\rangle^n + (1 - e^{-\beta_n t})\frac{\sum_{j=0}^{n-1}\kappa^n_j C_j(x_0)}{\beta_n}.\end{aligned}$$

The right hand side is a convex combination of two positive numbers. Hence we get

$$U^n_{x_0}(t) \leq \max\left\{\langle v, x_0\rangle^n, \frac{1}{\beta_n}\sum_{j=0}^{n-1}\kappa^n_j C_j(x_0)\right\} = C_n(x_0).$$

Taking supremum over $t \geq 0$ proves

$$\sup_{t\geq 0} U^n_{x_0}(t) \leq C_n(x_0).$$

Then part (B) follows from part (A).

We now prove part (C). Fix a $T > 0$ and a positive integer $n < (r_{\max} - 1)$. Then for any $j = 0, 1, \ldots, (n-1)$ we have

$$\lim_{t\to\infty}\int_0^T e^{-\beta_n(t-s)}U^j_{x_0}(s)ds \leq C_j(x_0)\lim_{t\to\infty}\int_0^T e^{-\beta_n(t-s)}ds = 0$$

and hence

$$\lim_{t\to\infty}\int_0^t e^{-\beta_n(t-s)}U^j_{x_0}(s)ds = \lim_{t\to\infty}\int_T^t e^{-\beta_n(t-s)}U^j_{x_0}(s)ds. \tag{S1.15}$$

The following relation certainly holds for $r=0$

$$\lim_{t\to\infty} U^r_{x_0}(t) = \hat{C}_r.$$

Assume that it holds for all integers r less than n. Then for any $j=0,1,\dots,(n-1)$

$$\frac{\inf_{t\in[T,\infty)} U^j_{x_0}(t)}{\beta_n} \le \lim_{t\to\infty}\int_T^t e^{-\beta_n(t-s)}U^j_{x_0}(s)ds \le \frac{\sup_{t\in[T,\infty)} U^j_{x_0}(t)}{\beta_n}.$$

Since $\lim_{t\to\infty} U^j_{x_0}(t) = \hat{C}_j$, using (S1.15) and letting $T\to\infty$ in the above relation shows that

$$\lim_{t\to\infty}\int_0^t e^{-\beta_n(t-s)}U^j_{x_0}(s)ds = \frac{\hat{C}_j}{\beta_n}.$$

Therefore we can conclude that

$$\begin{aligned}\lim_{t\to\infty} U^n_{x_0}(t) &= \lim_{t\to\infty}\left(e^{-\beta_n t}\langle v, x_0\rangle^n + \sum_{j=0}^{n-1}\kappa^n_j\int_0^t e^{-\beta_n(t-s)}U^j_{x_0}(s)ds\right)\\ &= \sum_{j=0}^{n-1}\kappa^n_j \lim_{t\to\infty}\int_0^t e^{-\beta_n(t-s)}U^j_{x_0}(s)ds\\ &= \frac{\sum_{j=0}^{n-1}\kappa^n_j\hat{C}_j}{\beta_n}\\ &= \hat{C}_n.\end{aligned}$$

Now part (C) follows from part (A). □

From the above theorem we can obtain uniform and asymptotic moment bounds for the process $(X_{x_0}(t))_{t\ge 0}$. For any positive integer r, let $\Psi^r(x_0,t)$ denote the r-th moment of $X_{x_0}(t)$. Then $\Psi^r(x_0,t)$ is a tensor of rank r whose entry at index $(i_1,\dots,i_r)\in\{1,\dots,d\}^r$ is given by

$$\Psi^r_{i_1\dots i_r}(x_0,t) = \sum_{y\in\mathcal{S}} y_{i_1}\dots y_{i_r}p_{x_0}(t,y) \tag{S1.16}$$

where $y=(y_1,\dots,y_d)$ and $p_{x_0}(t)$ is the distribution of $X_{x_0}(t)$. Then we have the following result on moment bounds.

Proposition S1.3. (Moment Bounds) *Assume that Condition* **DD** *holds. Pick a positive integer* $r<r_{\max}$ *and let* $C_r(x_0)$ *and* $\hat{C}_r$ *be the positive constants obtained in Theorem S1.2. Then for any index* $(i_1,\dots,i_r)\in\{1,2,\dots,d\}^r$ *we have*

$$\sup_{t\ge 0}\Psi^r_{i_1\dots i_r}(x_0,t) \le \frac{C_r(x_0)}{\prod_{j=1}^r v_{i_j}}$$

$$\text{and } \limsup_{t\to\infty}\Psi^r_{i_1\dots i_r}(x_0,t) \le \frac{\hat{C}_r}{\prod_{j=1}^r v_{i_j}} \text{ for all } x_0\in\mathcal{S}.$$

Proof. Note that if $v=(v_1,\dots,v_d)$ and $y=(y_1,\dots,y_d)\in\mathcal{S}$, then $y_i\le \|y\|_v/v_i$ for each i. Hence for any $(i_1,\dots,i_r)\in\{1,\dots,d\}^r$

$$\begin{aligned}\Psi^r_{i_1\dots i_r}(x_0,t) &= \sum_{y\in\mathcal{S}} y_{i_1}\dots y_{i_r}p_{x_0}(t,y)\\ &\le \frac{\sum_{y\in\mathcal{S}}\|y\|^r_v p_{x_0}(t,y)}{\prod_{j=1}^r v_{i_j}}\\ &= \frac{m^r_{x_0}(t)}{\prod_{j=1}^r v_{i_j}}.\end{aligned}$$

The result is now immediate from Theorem S1.2. □

The next lemma will be useful later.

Lemma S1.4. *Assume that Condition* **DD** *holds and let $C_r(x_0)$ and $\hat{C}_r$ be defined as above. If $c_5 = 0$ in* (S1.4b) *then there exist positive constants $C(x_0)$ and C such that for all positive integers r we have $C_r(x_0) \leq r!(C(x_0))^r$ and $\hat{C}_r \leq r!C^r$.*

Proof. Let $c = \max\{c_1, c_3, c_4\}$. Pick a $C > 0$ satisfying

$$C \geq \frac{1}{\log\left(1 + \frac{c_2}{c}\right)}. \tag{S1.17}$$

Observe that since $c_5 = 0$ we have $\beta_n = nc_2$ for each n. If $n \geq 2$ then

$$\begin{aligned}
\frac{1}{\beta_n}\sum_{j=0}^{n-1}\kappa_j^n j!C^j &= \frac{1}{\beta_n}\left[c_3\sum_{j=0}^{n-2}\frac{n!}{(n-j)!}C^j + c_4\sum_{j=0}^{n-2}\frac{jn!}{(n-j+1)!}C^j\right. \\
&\quad \left. + n!c_1C^{n-1} + \left(\frac{n!(n-1)}{2}\right)c_4C^{n-1}\right] \\
&\leq \frac{c}{\beta_n}\left[\sum_{j=0}^{n-1}\frac{n!}{(n-j)!}C^j + \sum_{j=0}^{n-1}\frac{jn!}{(n-j+1)!}C^j\right] \\
&\leq \frac{c}{\beta_n}\left[\sum_{j=0}^{n-1}\frac{n!}{(n-j)!}\left(1 + \frac{j+1}{n-j+1}\right)C^j\right] \\
&= \frac{c}{\beta_n}\left[\sum_{j=0}^{n-1}\frac{n!}{(n-j)!}\left(\frac{n+2}{n-j+1}\right)C^j\right] \\
&\leq \left(\frac{c(n+2)}{2\beta_n}\right)n!\left[\sum_{j=0}^{n-1}\frac{C^j}{(n-j)!}\right] \\
&= \left(\frac{c(n+2)}{2\beta_n}\right)n!C^n\left[\sum_{r=1}^{n}\frac{1}{r!C^r}\right] \\
&\leq \left(\frac{c}{c_2}\right)n!C^n\left(e^{\frac{1}{C}} - 1\right).
\end{aligned}$$

Therefore using (S1.17) we obtain

$$\frac{1}{\beta_n}\sum_{j=0}^{n-1}\kappa_j^n j!C^j \leq n!C^n\left(\frac{c}{c_2}\right)\left(e^{\log\left(1+\frac{c_2}{c}\right)} - 1\right) = n!C^n. \tag{S1.18}$$

We will prove this lemma by induction. Since $\log(1+x) \leq x$ for any $x \geq 0$ and C satisfies (S1.17), it must also satisfy

$$C \geq \frac{1}{\log\left(1 + \frac{c_2}{c}\right)} \geq \frac{c}{c_2} \geq \frac{c_1}{c_2} = \frac{\kappa_0^1}{c_2}.$$

Hence

$$\hat{C}_r \leq r!C^r \tag{S1.19}$$

holds for $r = 1$. Assume that it holds for all $r = 1, 2, \ldots, (n-1)$ for some integer $n \geq 2$. Then from (S1.18) we get

$$\hat{C}_n = \frac{1}{\beta_n}\sum_{j=0}^{n-1}\kappa_j^n\hat{C}_j \leq \frac{1}{\beta_n}\sum_{j=0}^{n-1}\kappa_j^n j!C^j \leq n!C^n.$$

This shows that (S1.19) holds for $r = n$. Hence we can conclude that (S1.19) is satisfied for all positive integers r. Defining $C(x_0) = \max\{\langle v, x_0\rangle, C\}$, one can verify in a similar way that

$$C_r(x_0) \leq (C(x_0))^r$$

for each positive integer r. This completes the proof of the lemma. □

Using Lemma S1.4 we obtain the following result.

Theorem S1.5. (Uniform Light-Tailedness) *Let $(X_{x_0}(t))_{t\geq 0}$ be the Markov process with generator $\mathbb{A}$ and initial state $x_0 \in \mathcal{S}$. Suppose that Condition* **DD** *holds with $c_5 = 0$. Then there exists a $\gamma > 0$ such that*

$$\sup_{t\geq 0} \mathbb{E}\left(e^{\gamma\|X_{x_0}(t)\|_v}\right) = \sup_{t\geq 0} \sum_{y\in\mathcal{S}} e^{\gamma\|y\|_v} p_{x_0}(t, y) < \infty.$$

Proof. For any integer $r \geq 1$ let $C_r(x_0)$ be as in Theorem S1.2. Let $C(x_0)$ be a positive constant such that $C_r(x_0) \leq r!(C(x_0))^r$ for every integer $r \geq 1$. Such a constant exists due to Lemma S1.4. From part (B) of Theorem S1.2 we have

$$\sup_{t\geq 0} \mathbb{E}\left(\|X_{x_0}(t)\|_v^r\right) \leq C_r(x_0) \leq r!C(x_0).$$

Observe that

$$\begin{aligned}
\sup_{t\geq 0} \mathbb{E}\left(e^{\gamma\|X_{x_0}(t)\|_v}\right) &\leq 1 + \sum_{r=1}^{\infty} \gamma^r \frac{\sup_{t\geq 0} \mathbb{E}\left(\|X_{x_0}(t)\|_v^r\right)}{r!} \\
&\leq 1 + \sum_{r=1}^{\infty} \gamma^r \frac{C_r(x_0)}{r!} \\
&\leq 1 + \sum_{r=1}^{\infty} (\gamma C(x_0))^r.
\end{aligned}$$

If we let $\gamma = 1/(2C(x_0))$ then

$$\sup_{t\geq 0} \mathbb{E}\left(e^{\gamma\|X_{x_0}(t)\|_v}\right) \leq 1 + \sum_{r=1}^{\infty} \frac{1}{2^r} < \infty.$$

This proves the theorem. □

S2 Ergodicity and Moment Convergence

Consider a Markov process $(X_{x_0}(t))_{t\geq 0}$ with state space $\mathcal{S}$ and initial state $x_0 \in \mathcal{S}$. Let $p_{x_0}(t) \in \mathcal{P}(\mathcal{S})$ be the distribution of $X_{x_0}(t)$, where $\mathcal{P}(\mathcal{S})$ is the space of all probability measures over $\mathcal{S}$. Such a Markov process is called *ergodic* if there exists a $\pi \in \mathcal{P}(\mathcal{S})$ such that

$$\lim_{t\to\infty} \|p_{x_0}(t) - \pi\|_{\text{TV}} = 0 \text{ for all } x_0 \in \mathcal{S}. \tag{S2.1}$$

Here π is the unique stationary distribution for the dynamics and $\|\cdot\|_{\text{TV}}$ denotes the *total-variation* norm over $\mathcal{P}(\mathcal{S})$ given by

$$\|\mu\|_{\text{TV}} = \sup_{A\subset\mathcal{S}} \mu(A).$$

If the convergence in (S2.1) is exponentially fast, then the process is called *exponentially ergodic.*

Meyn and Tweedie [11] have given a criterion for proving ergodicity for continuous time Markov processes. Their criterion involves checking certain *drift conditions* (called Foster-Lyapunov inequalities) based on the generator of the Markov process. In particular, if the state space $\mathcal{S}$ is countable and

irreducible for the Markov process $(X_{x_0}(t))_{t\geq 0}$ with generator $\mathbb{A}$, then the ergodicity of this process can be verified by finding a positive *norm-like*[1] function $V : \mathcal{S} \to \mathbb{R}$ such that for some $c_1, c_2 > 0$ we have

$$\mathbb{A}V(x) \leq c_1 - c_2 V(x) \text{ for all } x \in \mathcal{S}. \tag{S2.2}$$

In fact, the existence of such a function V shows that the process is exponentially ergodic. If Condition **DD** is satisfied for a positive vector v then the function V defined by $V(x) = \langle v, x\rangle$ satisfies (S2.2) due to condition (S1.4a). This gives our next result which is essentially a reformulation of Theorem 7.1 in [11].

Proposition S2.1. (Ergodicity) *Let $(X_{x_0}(t))_{t\geq 0}$ be the Markov process with generator $\mathbb{A}$ and initial state x_0. Assume that the state space $\mathcal{S}$ is irreducible and Condition* (S1.4a) *holds. Then this process is exponentially ergodic in the sense that there exists a unique stationary distribution $\pi \in \mathcal{P}(\mathcal{S})$ along with constants $B, c > 0$ such that for any $x_0 \in \mathcal{S}$*

$$\sup_{A\subset\mathcal{S}} |p_{x_0}(t, A) - \pi(A)| \leq Be^{-ct} \text{ for all } t \geq 0.$$

From now on we assume that the process $(X_{x_0}(t))_{t\geq 0}$ is ergodic with stationary distribution π. The next result is a simple consequence of ergodicity.

Proposition S2.2. *Assume that Condition* **DD** *holds. Let $f : \mathcal{S} \to \mathbb{R}$ be a function such that for some positive integer $r < (r_{\max} - 1)$ there exists a $C > 0$ satisfying*

$$|f(x)| \leq C(1 + \|x\|_v^r) \text{ for all } x \in \mathcal{S}. \tag{S2.3}$$

Then $\sum_{y\in\mathcal{S}} |f(y)|\pi(y) < \infty$ and for any $x_0 \in \mathcal{S}$

$$\lim_{t\to\infty} \mathbb{E}(f(X_{x_0}(t))) = \sum_{y\in\mathcal{S}} f(y)\pi(y). \tag{S2.4}$$

Furthermore for any $x_0 \in \mathcal{S}$, the following relation is satisfied with probability 1

$$\lim_{t\to\infty} \frac{1}{t}\int_0^t f(X_{x_0}(s))ds = \sum_{y\in\mathcal{S}} f(y)\pi(y). \tag{S2.5}$$

Proof. Suppose that f is a positive function. It suffices to prove the proposition in this case. We have assumed that for some positive integer $r < (r_{\max} - 1)$ there exists a $C > 0$ such that (S2.3) is satisfied. Let q be an integer satisfying $r < q < r_{\max}$ and let $\rho = q/r$. Note that $\rho > 1$. For all $x \in \mathcal{S}$ we have

$$(f(x))^\rho \leq C^\rho(1 + \|x\|_v^r)^\rho \leq C^\rho 2^\rho(1 + \|x\|_v^{r\rho}) = C^\rho 2^\rho(1 + \|x\|_v^q). \tag{S2.6}$$

Since $q < r_{\max}$, Theorem S1.2 shows that there exists a positive constant $\hat{C}_q$ such that

$$\limsup_{t\to\infty} \mathbb{E}\left(\|X_{x_0}(t)\|_v^q\right) \leq \hat{C}_q.$$

From (S2.6) we obtain that

$$\limsup_{t\to\infty} \mathbb{E}\left((f\left(X_{x_0}(t)\right))^\rho\right) \leq C^\rho 2^\rho(1 + \hat{C}_q). \tag{S2.7}$$

Note that ergodicity of the process $(X_{x_0}(t))_{t\geq 0}$ implies that for any set $A \subset \mathcal{S}$ if $\sup_{x\in A} f(x) < \infty$ then we have

$$\lim_{t\to\infty} \sum_{y\in A} f(y)p_{x_0}(t, y) = \sum_{y\in A} f(y)\pi(y). \tag{S2.8}$$

[1]A postive function $V : \mathcal{S} \to \mathbb{R}$ is called *norm-like* if the set $\{x \in \mathcal{S} : V(x) \leq c\}$ is compact for any $c > 0$.

Suppose that $\sum_{y\in\mathcal{S}} f(y)\pi(y) = \infty$. Then for any $M > 0$, there exists a finite set $A_M \subset \mathcal{S}$ such that $\sum_{y\in A_M} f(y)\pi(y) > M$. Therefore from (S2.8) we obtain

$$M < \sum_{y\in A_M} f(y)\pi(y) = \lim_{t\to\infty} \sum_{y\in A_M} f(y)p_{x_0}(t,y) \leq \limsup_{t\to\infty} \mathbb{E}\left(f(X_{x_0}(t))\right).$$

Let $M_0 = \limsup_{t\to\infty} \mathbb{E}\left(f(X_{x_0}(t))\right)$. Then M_0 is finite due to (S2.7). If we take M to be greater than M_0 then we have a contradiction. Hence $\sum_{y\in\mathcal{S}} f(y)\pi(y) < \infty$.

For $n = 1, 2, \ldots$ define a finite set

$$A_n = \{x \in \mathcal{S} : f(x) < nM_0\}$$

and let A_n^c denote the complement of A_n. Note $A_1 \subset A_2 \subset A_3 \subset \ldots$ and $\cup_{n=1}^{\infty} A_n = \mathcal{S}$. Using the monotone convergence theorem we obtain

$$\lim_{n\to\infty} \sum_{y\in A_n} f(y)\pi(y) = \sum_{y\in\mathcal{S}} f(y)\pi(y). \tag{S2.9}$$

From Markov's inequality we get

$$\begin{aligned}
\limsup_{t\to\infty} \mathbb{P}\left(f(X_{x_0}(t)) \notin A_n\right) &\leq \limsup_{t\to\infty} \mathbb{P}\left(f(X_{x_0}(t)) \geq nM_0\right) \\
&\leq \frac{\limsup_{t\to\infty} \mathbb{E}\left(f(X_{x_0}(t))\right)}{nM_0} \\
&\leq \frac{1}{n}.
\end{aligned}$$

Let $\mathbb{1}_{A_n^c}(\cdot)$ denote the indicator function of the set A_n^c. Let ρ' be the positive number satisfying $(1/\rho) + (1/\rho') = 1$. Using the Holder's inequality along with (S2.7) gives us

$$\begin{aligned}
\limsup_{t\to\infty} \sum_{y\in A_n^c} f(y)p_{x_0}(t,y) &= \limsup_{t\to\infty} \mathbb{E}\left(f(X_{x_0}(t))\mathbb{1}_{A_n^c}(X_{x_0}(t))\right) \\
&\leq \limsup_{t\to\infty} \left(\mathbb{E}\left(f(X_{x_0}(t))^{\rho}\right)\right)^{\frac{1}{\rho}} \left(\mathbb{E}\left(\mathbb{1}_{A_n^c}(X_{x_0}(t))\right)\right)^{\frac{1}{\rho'}} \\
&\leq \limsup_{t\to\infty} \left(\mathbb{E}\left(f(X_{x_0}(t))^{\rho}\right)\right)^{\frac{1}{\rho}} \left(\mathbb{P}\left(X_{x_0}(t) \notin A_n\right)\right)^{\frac{1}{\rho'}} \\
&\leq \frac{2C(1+\hat{C}_q)^{\frac{1}{\rho}}}{n^{\frac{1}{\rho'}}}.
\end{aligned}$$

Using this inequality along with (S2.8) we obtain

$$\begin{aligned}
\limsup_{t\to\infty} \mathbb{E}\left(f(X_{x_0}(t))\right) &= \limsup_{t\to\infty} \sum_{y\in\mathcal{S}} f(y)p_{x_0}(t,y) \\
&= \limsup_{t\to\infty} \left(\sum_{y\in A_n} f(y)p_{x_0}(t,y) + \sum_{y\in A_n^c} f(y)p_{x_0}(t,y)\right) \\
&\leq \limsup_{t\to\infty} \sum_{y\in A_n} f(y)p_{x_0}(t,y) + \limsup_{t\to\infty} \sum_{y\in A_n^c} f(y)p_{x_0}(t,y) \\
&\leq \sum_{y\in A_n} f(y)\pi(y) + \frac{2C(1+\hat{C}_q)^{\frac{1}{\rho}}}{n^{\frac{1}{\rho'}}}.
\end{aligned}$$

Letting $n \to \infty$ and using (S2.9) yields

$$\limsup_{t\to\infty} \mathbb{E}\left(f(X_{x_0}(t))\right) \leq \lim_{n\to\infty} \sum_{y\in A_n} f(y)\pi(y) = \sum_{y\in\mathcal{S}} f(y)\pi(y). \tag{S2.10}$$

Similarly one can show that

$$\liminf_{t\to\infty} \mathbb{E}\left(f(X_{x_0}(t))\right) \geq \sum_{y\in\mathcal{S}} f(y)\pi(y). \tag{S2.11}$$

Combining (S2.10) and (S2.11) proves (S2.4). The relation (S2.5) is just the law of large numbers for ergodic processes (see [10]). □

Using Proposition S2.2 we can prove convergence of moments of $(X_{x_0}(t))_{t\geq 0}$ to their stationary values. For any positive integer r, let Π^r denote the r-th moment of the stationary distribution π. Then Π^r is a tensor of rank r defined in the same way as $\Psi^r(x_0,t)$ (see (S1.16)), with $p_{x_0}(t,y)$ replaced by $\pi(y)$.

Theorem S2.3. (Moment Convergence) *Assume that Condition* **DD** *holds. Let r be any positive integer satisfying $r < (r_{\max} - 1)$. Then Π^r is finite (componentwise) and $\Psi^r(x_0,t) \to \Pi^r$ as $t \to \infty$.*

Proof. Let r be any positive integer satisfying $r < (r_{\max} - 1)$. Pick any index $(i_1,\dots,i_r) \in \{1,\dots,d\}^r$. Let $f : \mathcal{S} \to \mathbb{R}$ be given by $f(y) = y_{i_1}\dots y_{i_r}$, where $y = (y_1,\dots,y_d)$. If $v = (v_1,\dots,v_d)$ then $y_i \leq \|y\|_v / v_i$ for each i. Hence if $C = 1/(\prod_{j=1}^{r} v_{i_j})$ then for all $y \in \mathcal{S}$ we have

$$|f(y)| \leq C(1 + \|y\|_v^r).$$

Observe that

$$\Psi^r_{i_1,\dots,i_r}(x_0,t) = \sum_{y\in\mathcal{S}} f(y) p_{x_0}(t,y)$$

and

$$\Pi^r_{i_1,\dots,i_r} = \sum_{y\in\mathcal{S}} f(y)\pi(y).$$

To prove the result we have to show that $\Pi^r_{i_1,\dots,i_r} < \infty$ and $\Psi^r_{i_1,\dots,i_r}(x_0,t) \to \Pi^r_{i_1,\dots,i_r}$ as $t \to \infty$. The proof is now immediate from Proposition S2.2. □

From Proposition S2.2 we can also conclude that for any positive integer $r < (r_{\max} - 1)$ we have

$$\lim_{t\to\infty} \sum_{x\in\mathcal{S}} \|y\|_v^r p_{x_0}(t,y) = \sum_{y\in\mathcal{S}} \|y\|_v^r \pi(y).$$

Using part (C) of Theorem S1.2 we get

$$\sum_{y\in\mathcal{S}} \|y\|_v^r \pi(y) \leq \hat{C}_r. \tag{S2.12}$$

This allows us to prove the following result.

Theorem S2.4. (Light-Tailedness at stationarity) *Suppose that Condition* **DD** *holds with $c_5 = 0$. Then there exists a $\gamma > 0$ such that*

$$\sum_{y\in\mathcal{S}} e^{\gamma\|y\|_v}\pi(y) < \infty.$$

Proof. Note that if $c_5 = 0$ then $r_{\max} = \infty$ and (S2.12) holds for all positive integers r. Let C be a positive constant such that $\hat{C}_r \leq r! C^r$ for all integers $r \geq 1$. Such a constant exists due to Lemma S1.4.

Let $\gamma = 1/(2C)$. Due to (S2.12) we obtain

$$\begin{aligned}\sum_{y\in\mathcal{S}} e^{\gamma\|y\|_v}\pi(y) &\leq 1 + \sum_{r=1}^{\infty}\frac{\gamma^r}{r!}\sum_{y\in\mathcal{S}}\|y\|_v^r\pi(y)\\ &\leq 1 + \sum_{r=1}^{\infty}\frac{\gamma^r}{r!}\hat{C}_r\\ &\leq 1 + \sum_{r=1}^{\infty}(\gamma C)^r\\ &\leq 1 + \sum_{r=1}^{\infty}\frac{1}{2^r} < \infty.\end{aligned}$$

This proves the theorem. □

Using the analytical tools developed in the previous sections, several general results can be stated for the class of unimolecular reaction networks and bimolecular reaction networks. In what follows, when we say that a moment is bounded, we mean that it is bounded uniformly in time. This can be established using Theorem S1.2 once Condition **DD** is verified. Furthermore, when we say that a moment is globally converging, we mean that it converges to its equilibrium value as time tends to infinity, irrespective of the initial state x_0. Once, Condition **DD** is verified, this can established using Theorem S2.3.

S3 Results on unimolecular reaction networks

We now discuss results on unimolecular reaction networks. As shown in the paper, these networks exhibit certain interesting properties. For instance, the boundedness of the all the moments can be judged from the feasibility of a linear program. Moreover this feasibility criterion also proves that the underlying (irreducible) Markov process representing the reaction network is ergodic.

For unimolecular networks, condition (S1.4a) can be reformulated as

$$p(c_1, c_2, v, x) := x^{\intercal}(A + c_2 I)v + b^T v - c_1 \leq 0 \tag{S3.1}$$

for all $x \in \mathbb{R}^d_{\geq 0}$. Here, the matrix A is Metzler[2] and the vector b is nonnegative. Linearity of the propensity functions implies that condition (S1.4b) can always be satisfied with $c_5 = 0$, for a suitable choice of positive constants c_3 and c_4. Hence we do not consider this condition further in this section. The rest of this section is devoted to deriving theoretical and computational results for characterizing the existence of $v > 0$ such that (S3.1) holds and, when this is the case, finding (sub)optimal values for c_1 and c_2 in order to compute asymptotic moment bounds and infer ergodicity of the Markov process.

S3.1 Proof of Proposition 7 and extensions to uncertain networks

We prove here Proposition 7 in the main text, which is recalled below for convenience.

Proposition S3.1 (Nominal ergodicity). *Let us consider the general unimolecular reaction network (15) and assume that the state-space of the underlying Markov process is irreducible. Let the matrices $A \in \mathbb{R}^{d\times d}$ and $b \in \mathbb{R}^d_{\geq 0}$, $||b|| \neq 0$, be further defined as*

$$\sum_{n=1}^{K}\lambda_n(x)\langle v, \zeta_n\rangle = x^{\intercal}Av + b^{\intercal}v. \tag{S3.2}$$

Then, the following statements are equivalent:

1. *The matrix A is Hurwitz-stable, i.e. it has all its eigenvalues in the open left half-plane.*
2. *There exists a positive vector $v \in \mathbb{R}^d$ such that $Av < 0$.*

[2] A square real matrix is Metlzer when all its off-diagonal elements are nonnegative.

Moreover, when one of the above statements holds, the Markov process describing the reaction network is exponentially ergodic and all the moments are bounded and globally converging. ◇

Proof. To prove the equivalence between statements 1) and 2), it is enough to note that the matrix A is Metzler. Then, from linear positive systems theory [8], the result follows. If one of the statements holds, then there exist $v > 0$ and c_2 such that $Av \leq -c_2 v$. Choosing then $c_1 = b^{\intercal} v$ shows exponential ergodicity of the process. Noticing finally that the Condition **DD** holds with $c_5 = 0$ proves that all the moments are bounded and globally converging.

□

Now assume that the Metzler matrix A in (S3.1) depends on a vector $\delta \in [-1, 1]^{\eta}$ where $\eta \in \mathbb{N}$ is the number of distinct uncertain parameters. We write this matrix as $A(\delta)$. Several methods can be used in order to deal with this case with uncertainities in the propensity functions.

Exact method Suppose that there exists a matrix $A_+ \in \mathbb{R}^{d\times d}$ satisfying the following properties:

1. $A(\delta) \leq A_+$ (in the componentwise sense) for all $\delta \in [-1, 1]^{\eta}$
2. There exists a $\delta^* \in [-1, 1]^{\eta}$ such that $A_+ = A(\delta^*)$.

Note that such a matrix A_+ may not exist, especially when some entries are not independent. However, when A_+ exists we have the following result.

Theorem S3.2 (Robust ergodicity)**.** *Let us consider the general unimolecular reaction network (15) described by the uncertain Metzler matrix $A(\delta)$ that we assume to admit the upper-bound A_+ defined above. Assume further that the state-space of the underlying Markov process is irreducible for all uncertain parameter values $\delta \in [-1, 1]^{\eta}$. Then, the following statements are equivalent:*

1. *The matrix $A(\delta)$ is Hurwitz-stable for all $\delta \in [-1, 1]^{\eta}$.*
2. *The matrix A_+ is Hurwitz-stable.*
3. *There exists a positive vector $v \in \mathbb{R}^d$ such that $A_+ v < 0$.*

Moreover, when one of the above statements holds, the Markov process describing the reaction network is robustly exponentially ergodic and and all the moments are bounded and globally converging. ◇

Proof. The proof relies on the fact that for two Metzler matrices $M_1, M_2 \in \mathbb{R}^{n\times n}$ such that $M_2 \geq M_1$ componentwise, we have that $\lambda_F(M_2) \geq \lambda_F(M_1)$ where $\lambda_F(\cdot)$ is the Frobenius eigenvalue [1]. Therefore, assuming that A_+ defined in the sense of (S3.3) exists, proving that A_+ is Hurwitz-stable implies that $A(\delta)$ is Hurwitz-stable for all $\delta \in [-1, 1]^{\eta}$. Conversely, having $A(\delta)$ Hurwitz-stable for all $\delta \in [-1, 1]^{\eta}$ implies that A_+ is Hurwitz-stable as well since there exists at least one $\delta \in [-1, 1]^{\eta}$ for which we have $A_+ = A(\delta)$. □

Approximate method When A_+ is not defined, it is possible to derive a conservative criterion using the matrix

$$A_{\pm} := \sup_{\delta\in[-1,1]^{\eta}} \{A(\delta)\} \tag{S3.3}$$

where the supremum is again taken in the in the componentwise sense. Note that, in this case, the matrix $A_{\pm}$ is always well-defined and we have that $A\pm = A_+$ when A_+ exists. The use of $A_{\pm}$ leads to the following result.

Theorem S3.3 (Robust ergodicity)**.** *Let us consider the general unimolecular reaction network (15) described by the uncertain Metzler matrix $A(\delta)$. Assume further that the state-space of the underlying Markov process is irreducible for all uncertain parameter values $\delta \in [-1, 1]^{\eta}$. Then, the following statements are equivalent:*

1. *The matrix $A_{\pm}$ is Hurwitz-stable.*
2. *There exists a positive vector $v \in \mathbb{R}^d$ such that $A_{\pm} v < 0$.* ◇

Then, the matrix $A(\delta)$ is Hurwitz-stable for all $\delta \in [-1,1]^\eta$ and the stochastic reaction network is exponentially ergodic and all the moments are bounded and globally converging.

Proof. The proof follows along the same lines as the first robustness result. $\square$

Note, however, that the Hurwitz-stability condition on $A_\pm$ is only sufficient. It is also possible to merge these two approaches by first choosing a maximal subset of parameters for which A_+ is well-defined and then consider the remaining parameters $\delta' \in [-1,1]^{\eta'}$, $\eta' < \eta$ in a robust analysis setting by looking for a constant vector $v > 0$ such that $A_+(\delta')v < 0$. A parameter dependent $v(\delta')$ may also be considered, at the expense of computational complexity and poor scalability. Finally, the method of [3] can be used as well in order to explicitly consider the structure of the parameter dependence of the matrix $A(\delta)$.

Example S3.4. *Let us consider the Metzler matrix*

$$A(\delta) = \begin{bmatrix} -1 & 0 \\ 2+\delta & -3-\delta \end{bmatrix}$$

where $\delta \in [-1,1]$. This matrix does not admit an upper-bound A_+ since the $(2,1)$ entry is maximum when $\delta = 1$ while the (2,2) entry is maximum when $\delta = -1$. However, the corresponding $A_\pm$ matrix is given by

$$A_\pm = \begin{bmatrix} -1 & 0 \\ 3 & -2 \end{bmatrix}.$$

Note that since the matrix $A_\pm$ is Hurwitz-stable, then $A(\delta)$ is also Hurwitz-stable for all $\delta \in [-1,1]$. If, however, we modify $A(\delta)$ so that the (1,2) entry is now equal to 1, then the matrix $A(\delta)$ is still Hurwitz-stable for all $\delta \in [-1,1]$ but $A_\pm$ is not.

S3.2 Computing an optimal value for $\widehat{C}_1$ when the vector v is given

Assume now that we want to find the minimum value for the asymptotic first-order moment bound of $\langle v, X(t)\rangle$ given by $\widehat{C}_1 = c_1/c_2$ in Theorem S1.2. Let $v > 0$ be given. In this case, finding an optimal value for the ratio c_1/c_2 can be cast as the following optimization problem:

$$\begin{array}{ll} \min\limits_{c_1,c_2} \widehat{C}_1 := \dfrac{c_1}{c_2} & \text{s.t.} \\ & c_1, c_2 > 0 \\ & p(c_1,c_2,v,x) \le 0,\ \forall x \in \mathbb{R}^N_{\ge 0}. \end{array} \tag{S3.4}$$

Since the polynomial $p(c_1,c_2,v,x)$ is affine in x, the above optimization problem is equivalent to

$$\begin{array}{ll} \min\limits_{(c_1,c_2)\in\mathbb{R}^2} \widehat{C}_1 & \text{s.t.} \\ & c_1, c_2 > 0 \\ & (A + c_2 I)v \le 0 \\ & b^T v - c_1 \le 0. \end{array} \tag{S3.5}$$

The above optimization problem with rational cost can be equivalently turned into a linear program, as presented below:

Theorem S3.5. *The optimal solution z^* of the linear program*

$$\begin{array}{ll} \max\limits_{z\in\mathbb{R}} z & s.t. \\ & z > 0 \\ & (zI + A)v \le 0 \end{array} \tag{S3.6}$$

is related to the solution $\widehat{C}_1^*$ of the program (S3.5) by $\widehat{C}_1^* = \dfrac{b^{\mathsf{T}} v}{z^*}$.

Proof. The optimization problem (S3.4) is a linear fractional programming problem [2, pp. 151]. Using the change of variables $y_1 = c_1/c_2$, $y_2 = 1/c_2$ (see [2, pp. 151]), we can reformulate it as the following equivalent linear programming problem

$$\min_{(y_1,y_2)\in\mathbb{R}^2} y_1 \quad \text{s.t.} \quad \begin{array}{l} v + Avy_2 \leq 0 \\ -y_1 + y_2 b^\intercal v \leq 0 \\ y_2 \geq 0 \end{array} \tag{S3.7}$$

and we have $y_1^* = \widehat{C}_1^*$.

Noting that minimizing over $y_1 > 0$ according to the second constraint is equivalent to saying that $y_1^* = b^\intercal v y_2^*$, we obtain the optimization problem

$$\min_{y_2\in\mathbb{R}} y_2 \quad \text{s.t.} \quad \begin{array}{l} (I + y_2 A)v \leq 0 \\ y_2 \geq 0 \end{array} \tag{S3.8}$$

and we have $\widehat{C}_1^* = b^\intercal v y_2^*$. The problem is obviously not feasible for $y_2 = 0$ since $v > 0$ by assumption. Therefore, letting $z := 1/y_2$ yields the result. □

Remark S3.6. *The computational complexity of the optimization problem* (S3.6) *scales linearly with respect to the number of species. The number of variables is 1 and the number of constraints is $d + 1$. Note that the number of reactions does not have any impact on the complexity of the optimization problem.*

S3.3 Computing an optimal value for $\widehat{C}_1$ when the vector v contains some decision variables

In this case, the problem involves bilinear constraints and a globally convergent bisection procedure can be used to maximize c_2 (but may not globally minimize c_1/c_2). In the following, the vector $\theta \in \mathbb{R}^\ell_{>0}$, $\ell \leq d$ is used to denote the decision variables. In this case, we denote by $v(\theta)$ the vector v depending on the decision vector θ.

S3.3.1 Some entries of v are fixed

We assume here that $\ell < d$. The optimization problem that needs to be solved in this case is given by

$$\max_{(z,\theta)\in\mathbb{R}^{\ell+1}} z \quad \text{s.t.} \quad \begin{array}{l} z > 0 \\ v_i(\theta) > \epsilon_i, i = 1, \ldots, \ell \\ (zI + A)v(\theta) \leq 0 \end{array} \tag{S3.9}$$

where the ϵ_i's are lower bounds on the $v_i(\theta)$'s in order to avoid 0 values (if required). This problem can be solved using a bisection algorithm and we have

$$\widehat{C}_1^* \leq \frac{b^\intercal v(\theta^*)}{z^*}. \tag{S3.10}$$

In general equality does not hold in (S3.10). However when $b^\intercal v(\theta)$ does not depend on θ, the computed optimum is global for the specific choice of $v(\theta)$ and constraints on θ.

Remark S3.7 (Complexity)**.** *In this optimization problem, the number of constraints is $d + \ell + 1$ while the number of variables is $\ell + 1$. The computational complexity therefore grows linearly with respect to the number of species.*

S3.3.2 v has to be fully determined

When $\ell = d$, the vector $v = \theta$ has to be fully determined. In this case, a problem may occur when the matrix A is reducible[3]. If this happens, the optimal solution for v may contain 0 entries, and therefore this solution cannot be used for our subsequent analysis. Nevertheless, such a solution gives some insights on the optimal value $c_2^* = z^*$.

Perron-Frobenius Theorem Since A in (S3.1) is a Metzler matrix, Perron-Frobenius theory can be applied to determine the suitable values for v and z such that $(zI + A)v = 0$ holds. Note that this problem is an eigenvalue problem.

Theorem S3.8 (Perron-Frobenius Theorem). *Let $M \in \mathbb{R}^{n\times n}$ be an irreducible Metzler matrix and let $\tau > 0$ be such that $\tau I + M$ is nonnegative and has spectral radius ρ. Then,*

1. *the number ρ is positive and is an eigenvalue of $\tau I + M$;*
2. *the eigenvalue ρ is simple;*
3. *the matrix $M + \tau I$ has a right-eigenvector with positive entries;*
4. *ρ is the only eigenvalue having a positive eigenvector.*

In order to apply this result to our problem, set $\tau = -\min\{0, a_{11}, \ldots, a_{dd}\} \geq 0$ to have $A + \tau I$ nonnegative. The optimal value z^* is then simply given by $z^* = \tau - \rho$ and we have $(z^* I + A)v^* = 0$ where v^* is the positive right-eigenvector associated with the eigenvalue ρ of $\tau I + A$.

An example where v does not contain any 0 entry Let A and b be given by

$$A = \begin{bmatrix} -2 & 1 \\ 1 & -3 \end{bmatrix}, \ b = \begin{bmatrix} 1 \\ 0 \end{bmatrix}. \tag{S3.11}$$

Clearly, A is irreducible. Choosing $\tau = 3$, we get that $\rho = \dfrac{1+\sqrt{5}}{2}$ and therefore

$$z^* = \frac{5-\sqrt{5}}{2} \text{ and } v^* = \begin{bmatrix} \left(\dfrac{1+\sqrt{5}}{2}\right) \\ 1 \end{bmatrix}.$$

In this case, $c_1 = v_1 = \dfrac{1+\sqrt{5}}{2}$ and then we have

$$\lim_{t\to\infty} \mathbb{E}\left[(1+\sqrt{5})X_1(t) + 2X_2(t)\right] \leq 1 + \frac{3}{5}\sqrt{5} \tag{S3.12}$$

where $(X_1(t), X_2(t))_{t\geq 0}$ is the Markov process describing the reaction network associated with the matrices (S3.11). Let

$$E(t) := \begin{bmatrix} \mathbb{E}[X_1(t)] \\ \mathbb{E}[X_2(t)] \end{bmatrix}$$

be the vector of first-order moments. Then, we have that

$$\dot{E}(t) = A^\intercal E(t) + b, \ E(0) = E_0. \tag{S3.13}$$

The above dynamical system can be used to check the tightness of the bound in (S3.12). To this aim, we compute the equilibrium point of the above system which is given by

$$E^* = -A^{-\intercal} b = \frac{1}{5}\begin{bmatrix} 3 \\ 1 \end{bmatrix}. \tag{S3.14}$$

Substituting then the equilibrium solution (S3.14) in the left-hand side of (S3.12), we obtain that

$$(1+\sqrt{5})E_1^* + 2E_2^* = 1 + \frac{3}{5}\sqrt{5}$$

which shows the exactness of the computed upper-bound in this case.

[3]A matrix is reducible if and only if it can be placed into a block upper-triangular form by simultaneous row/column permutations. In addition, a matrix is reducible if and only if its associated digraph is not strongly connected.

An example where v contains 0 entries When A is reducible, there may not be a positive v^* such that $(z^*I + A)v^* = 0$ holds, as shown in the following example.

Example S3.9. *Assume that A and b are given by*

$$A = \begin{bmatrix} -1 & 1 \\ 0 & -2 \end{bmatrix}, \; b = \begin{bmatrix} 1 \\ 0 \end{bmatrix}.$$

Clearly, A is reducible and the optimal z is given by $z^* = 1$ but the corresponding v is given by $v^* = \begin{bmatrix} 1 & 0 \end{bmatrix}^\intercal$ which contains a 0 entry.

In such case, there exist two possibilities: either pick a suboptimal z^* and solve the problem in an analytical way, or use an optimization algorithm. We describe both these approaches below.

Algorithmic solution A computational way to solve the '0-entry problem' relies on the following optimization problem where positive lower bounds ϵ_i are imposed on the components of v:

$$\begin{array}{ll} \max\limits_{(z,v)\in\mathbb{R}^{d+1}} z & \text{s.t.} \\ & z > 0 \\ & v_i > \epsilon_i, i = 1, \dots, d \\ & (zI + A)v \leq 0. \end{array} \tag{S3.15}$$

This problem can be solved using a bisection algorithm and we have

$$\widehat{C}_1^* \leq \frac{b^\intercal v^*}{z^*}. \tag{S3.16}$$

Analytical solution The idea here is to simply pick a small $\varepsilon > 0$ for which we have $((z^* - \varepsilon)I + A)v_\varepsilon \leq 0$ for some $v_\varepsilon > 0$, and then solve the problem analytically. In the case of Example S3.9, if we pick a small $\varepsilon \in (0, 1)$, there will exist a corresponding v_ε such that $((z^* - \varepsilon)I + A)v_\varepsilon \leq 0$ holds. A suitable v_ε is given by $v_\varepsilon = \begin{bmatrix} \xi/\varepsilon & \xi \end{bmatrix}$ for some $\xi > 0$. The optimal choice for c_1 is therefore $c_1^* = \xi/\varepsilon$ in this case. Looking at the ratio $c^* := c_1^*/c_2^*$, we find that

$$c^* = \frac{\xi}{\varepsilon(1 - \varepsilon)} \tag{S3.17}$$

and therefore

$$\lim_{t\to\infty} \mathbb{E}\left[\frac{\xi}{\varepsilon} X_1(t) + \xi X_2(t)\right] \leq \frac{4\xi}{\varepsilon(1 - \varepsilon)}. \tag{S3.18}$$

The right-hand side is minimum when $\varepsilon = 1/2$, and this yields

$$\lim_{t\to\infty} \mathbb{E}[2\xi X_1(t) + \xi X_2(t)] \leq 4\xi, \tag{S3.19}$$

and hence

$$\lim_{t\to\infty} \mathbb{E}[2X_1(t) + X_2(t)] \leq 4. \tag{S3.20}$$

Again, we can check the validity of the bound by looking at the stationary solution of the dynamical system representing the dynamics of the first-order moments:

$$\dot{E}(t) = A^\intercal E(t) + b, \; E(0) = E_0 \tag{S3.21}$$

given by

$$E^* = -A^{-\intercal} b = \begin{bmatrix} 1 \\ 1/2 \end{bmatrix}. \tag{S3.22}$$

Substituting the stationary solution in (S3.20), shows that the bound 4 is not tight since $2E_1^* + E_2^* = 2.5 < 4$.

The take-away message here is that minimizing (S3.17) does not always minimize the gap between the right-hand side and the left-hand side of (S3.18). This gap, moreover, clearly depends on the choice of v. Note also that minimizing such a gap is generally not possible since the equilibrium values may not be known (e.g. in the bimolecular case for instance).

S4 Results on bimolecular reaction networks

When bimolecular networks are considered, the condition (S1.4a) can be reformulated as

$$p(c_1, c_2, v, x) = x^\intercal M(v)x + x^\intercal (A + c_2 I)v + b^\intercal v - c_1 \leq 0, \;\; x \in \mathbb{R}^d_{\geq 0}. \tag{S4.1}$$

The presence of the quadratic term $x^\intercal M(v)x$ clearly complicates the analysis. Several approaches can therefore be considered to take care of this term:

1. For some networks, the quadratic terms can be eliminated from the constraints through an appropriate choice for v, simplifying the subsequent analysis. For these networks, boundedness of all moments follows from the boundedness of the first-order moments. As in the previous section, ergodicity can be proved using simple results from linear algebra or by solving linear programming problems.

2. For some other networks, the quadratic terms cannot be eliminated but the condition (S4.1) can be exactly cast as a linear programming problem. Ergodicity holds but boundedness of all the moments does not hold in general.

3. In all the other cases, we will have to deal with a copositivity problem (NP-complete problem [12]) and will have to rely upon conservative LP and SDP relaxations, some of them being asymptotically exact, yet very complex numerically. In this case, again, ergodicity will hold but boundedness of all the moments is not guaranteed.

Only the two first cases are theoretically developed in the current paper. The latter one will only be illustrated in Section S11 through an example. The full theory of the latter case will be addressed in a future paper.

S4.1 Proof of Propositions 10 and 11

To address Case 1, it is convenient to define S_q as the restriction of the stoichiometry matrix S to bimolecular reactions. Define

$$\mathcal{N}_q := \left\{ v \in \mathbb{R}^d : \;\; v > 0, \;\; v^\intercal S_q = 0 \right\}$$

which consists of positive vectors in the left null-space of S_q. Then we have the following result (corresponding to Proposition 10 in the paper).

Proposition S4.1 (Nominal ergodicity for bimolecular networks)**.** *Let us consider the general bimolecular reaction network (18) and assume that the state-space of the underlying Markov process is irreducible. Assume further that there exists a vector $v \in \mathcal{N}_q$ such that $Av < 0$.*

Then, the stochastic bimolecular reaction network (18) is ergodic and all the moments are bounded and globally converging. ◇

Proof. The proof follows from the fact that if $v \in \mathcal{N}_q$, then (S4.1) reduces to

$$p(c_1, c_2, v, x) = x^\intercal (A + c_2 I)v + b^\intercal v - c_1 \leq 0 \tag{S4.2}$$

which is identical as in the unimolecular case. Then, the existence of $v \in \mathcal{N}_q$, $c_1, c_2 > 0$ such that the above inequality holds for all $x \in \mathbb{R}^d_{\geq 0}$ proves ergodicity. The boundedness and global convergence of all the moments follows from Corollary S2.3 and the fact that c_5 can be set to 0 in (S1.4b). □

The critical role of the space $\mathcal{N}_q$ imposes a restriction on the number of bimolecular reactions. The non-emptiness of $\mathcal{N}_q$ is equivalent to the existence of a conservation law for all the bimolecular reactions, i.e. a linear combination of all the species whose value is preserved over time when considering only bimolecular reactions. Note that this definition extends to more general mass-action kinetics as well. A necessary condition for the non-emptiness of $\mathcal{N}_q$ is that S_q is not full-row rank. Note also that when the number of bimolecular reactions increases, the dimension of the space $\mathcal{N}_q$ decreases. This progressively restricts the possible choices for v, potentially disabling approach to yield conclusive results.

When $\mathcal{N}_q$ is empty, we may ask whether it is possible to exactly verify (S4.1) using linear programming techniques. First note that if $M(v)$ in (S4.1) is such that $x^\intercal M(v)x \leq 0$ holds for all $x \geq 0$, then the

condition (S4.1) will hold provided that $Av < 0$. Checking whether a symmetric matrix $M(v)$ verifies this property is known as the coposivity (or conegativity in this case) problem which is NP-complete [12]. However, when the matrix $M(v)$ exhibits a certain sparsity structure, then checking copositivity exactly reduces to a tractable linear programming problem. This sparsity structure is defined below:

Definition S4.2. *We say that a symmetric real matrix $W = [w_{ij}]$ is of class $\mathcal{S}_I$ if $w_{ii}w_{ij}w_{jj} = 0$ for all $i, j = 1, \ldots, d$. A network is said to be a bimolecular $\mathcal{S}_I$ network if its matrix $M(v)$ defined in* (S4.1) *is of class $\mathcal{S}_I$.*

For a matrix exhibiting such a sparsity structure, we have the following result:

Lemma S4.3. *Let us consider a real symmetric matrix $W = [w_{ij}] \in \mathbb{R}^{d\times d}$ is of class $\mathcal{S}_I$. Then, the following statements are equivalent:*

1. *The matrix W is copositive, i.e. $x^T Wx \geq 0$ for all $x \geq 0$.*
2. *The matrix W is nonnegative.*

Proof. The proof that W nonnegative implies that the quadratic form is copositive is straightforward. The converse is less obvious. Assume that the matrix W is copositive and of class $\mathcal{S}_I$, then the quadratic polynomial $q(x) := x^T Wx$ does not have terms of the form $w_{ii}x_i^2 + 2w_{ij}x_ix_j + w_{jj}x_j^2$. Letting $x_i > 0$ and $x_j = 0$ for all $j \neq i$, we get that $q(x) = w_{ii}x_i^2$. Copositivity then implies that $w_{ii} \geq 0$.

What remains to be proved now is the nonnegativity of the w_{ij}'s. To show this, assume w.l.o.g. that x_i and x_j are involved as $w_{ii}x_i^2 + 2w_{ij}x_ix_j$ and set all the other entries of x to zero. Then $q(x) = w_{ii}x_i^2 + 2w_{ij}x_ix_j$. It is clear that since x_i and x_j are independent, then x_i^2 and x_ix_j are independent as well and thus copositivity of W implies that $w_{ij} \geq 0$. This argument can be repeated for all the terms in $q(x)$, thereby proving the result. □

The above result gives a way to efficiently check whether the matrix $M(v)$ is conegative.

Proposition S4.4 (Nominal ergodicity for bimolecular networks)**.** *Let us consider the general bimolecular reaction network (18) and assume that the state-space of the underlying Markov process is irreducible. Assume further that there exists a vector $v > 0$ such that $Av < 0$ and $M(v)$ is conegative.*

Then, the stochastic bimolecular reaction network (18) is ergodic and all the moments up to order $\lfloor 1 + 2c_2/c_5\rfloor - 2$ are bounded and globally converging. ◇

Proof. As before, the goal is to show that (S4.1) holds for some $c_1, c_2 > 0$. It is immediate to see that the under the assumptions of the theorem, we have the existence of $c_2 > 0$ such that $x^\intercal(A+c_2I)v+x^\intercal M(v)x \leq 0$ for all $x \geq 0$, and therefore ergodicity holds. Moreover, since the reaction network is bimolecular the term c_5 in (S1.4b) is nonzero, from Theorem (2) and formula (S1.11), we can state that all the moments up to $\lfloor 1 + 2c_2/c_5\rfloor - 2$ are bounded and globally converging. □

In case, one can find a vector $v > 0$ such that $M(v)$ is negative definite, then the constraint on the stability of the matrix A can be relaxed, as demonstrated by the following proposition:

Proposition S4.5 (Nominal ergodicity for bimolecular networks)**.** *Let us consider the general bimolecular reaction network (18) and assume that the state-space of the underlying Markov process is irreducible. Assume further that there exists a vector $v > 0$ such that $M(v)$ is negative-definite.*

Then, the stochastic bimolecular reaction network is ergodic and all the moments up to order $\lfloor 1 + 2c_2/c_5\rfloor - 2$ are bounded and globally converging. ◇

Proof. First note that we have the following equality

$$\begin{array}{rcl}b^T v + x^\intercal Av + x^\intercal M(v)x & = & \left(x + \dfrac{1}{2}M(v)^{-1}Av\right)^\intercal M(v)\left(x + \dfrac{1}{2}M(v)^{-1}Av\right)\\ & & -\dfrac{1}{4}v^\intercal A^\intercal M(v)^{-1}Av + b^T v.\end{array} \tag{S4.3}$$

The condition (S1.4a) can hence be reformulated as

$$\left(x + \frac{1}{2}M(v)^{-1}\tilde{A}v\right)^\intercal M(v)\left(x + \frac{1}{2}M(v)^{-1}\tilde{A}v\right) - \frac{1}{4}v^\intercal\tilde{A}^\intercal M(v)^{-1}\tilde{A}v \leq 0 \tag{S4.4}$$

where $\tilde{A} = A + c_2 I$ and we have set that $c_1 = b^T v$. Clearly, the inequality is not satisfied for all $x \in \mathbb{N}_0^d$ but is satisfied for all $x \in \mathbb{N}_0^d$ outside the set

$$\mathcal{E} := \left\{ x \in \mathbb{R}^d : \left(x + \frac{1}{2} M(v)^{-1} \tilde{A} v \right)^\intercal M(v) \left(x + \frac{1}{2} M(v)^{-1} \tilde{A} v \right) \geq \frac{1}{4} v^\intercal \tilde{A}^\intercal M(v)^{-1} \tilde{A} v \right\}. \tag{S4.5}$$

This implies that c_1 can be adapted to such that the inequality (S1.4a) is satisfied for all $x \in \mathbb{N}_0^d$. □

S4.2 Optimization problem corresponding to Proposition S4.1

When v contains $0 < \ell \leq d$ distinct decision variables, denoted by $\theta \in \mathbb{R}^\ell$, the following program can be used to check the conditions of Proposition S4.1:

$$\begin{array}{rl} \max\limits_{(z,\theta) \in \mathbb{R}^{\ell+1}} & z \\ \text{s.t.} & z > 0 \\ & v_i(\theta) > \epsilon, \ i = 1, \ldots, \ell \\ & (zI + A) v(\theta) \leq 0 \\ & v(\theta)^\intercal S_q = 0. \end{array} \tag{S4.6}$$

Moreover, when the optimization problem is feasible, we have that

$$\widehat{C}_1^* \leq b^\intercal \frac{v(\theta^*)}{z^*} \tag{S4.7}$$

where (z^*, v^*) is a global minimizer of the problem. Note that the additional equality constraint $v(\theta)^\intercal S_q = 0$ imposes that $v(\theta) \in \mathcal{N}_q$.

S4.3 Details on the example about the non-ergodic process with unbounded moments

Let us consider now the following reaction network with mass-action kinetics:

$$\begin{array}{rcl} \emptyset & \xrightarrow{1} & \boldsymbol{S_1} \\ \emptyset & \xrightarrow{1} & \boldsymbol{S_2} \\ \boldsymbol{S_1} + \boldsymbol{S_2} & \xrightarrow{1} & \emptyset. \end{array} \tag{S4.8}$$

Let $\kappa \in \mathbb{R}_{\geq 0}^2$ be the vector of concentrations that denotes the state for the deterministic model. Then we have that

$$\begin{array}{rcl} \dot{\kappa}_1(t) & = & 1 - \kappa_1(t)\kappa_2(t) \\ \dot{\kappa}_2(t) & = & 1 - \kappa_1(t)\kappa_2(t). \end{array} \tag{S4.9}$$

Assume that $\kappa_2(0) - \kappa_1(0) = \alpha$, for some $\alpha \in \mathbb{R}$, then clearly $\kappa_2(t) - \kappa_1(t) = \alpha$ for all $t \geq 0$. Moreover, the unique positive equilibrium point for this class of initial conditions is given by

$$\kappa_1^* = \frac{1}{2}\left(-\alpha + \sqrt{\alpha^2 + 4} \right) \text{ and } \kappa_2^* = \frac{1}{2}\left(\alpha + \sqrt{\alpha^2 + 4} \right). \tag{S4.10}$$

To show that this equilibrium point is globally exponentially stable let

$$\sigma := (\kappa_1 - \kappa_1^*)^2 + (\kappa_2 - \kappa_2^*)^2. \tag{S4.11}$$

Since $\kappa_2(t) = \kappa_1(t) + \alpha$ for all $t \geq 0$, we get that

$$\sigma = 2(\kappa_1 - \kappa_1^*)^2. \tag{S4.12}$$

Differentiating σ yields

$$\begin{array}{rcl} \dot{\sigma} & = & -2\left[\kappa_1 + \frac{1}{2}\left(\alpha + \sqrt{\alpha^2 + 4} \right) \right] \sigma \\ & \leq & -\left(\alpha + \sqrt{\alpha^2 + 4} \right) \sigma \end{array} \tag{S4.13}$$

where the last inequality has been obtained using the fact that $\kappa_1(t) \geq 0$. This relation shows that $\sigma(t) \to 0$ exponentially fast as $t \to \infty$, which proves global exponential stability of the unique fixed point of the system in the deterministic setting.

We now examine the stochastic model for the reaction network. Let $X(t) = (X_1(t), X_2(t))$ be the state at time t of the Markov process describing the reaction network (S4.8). Assume that $X_1(0) - X_2(0) = \alpha$ for some $\alpha \in \mathbb{Z}$. Let $\mathbb{A}$ be the generator of the Markov process and let $F_1(x) = x_1 - x_2$ for $x = (x_1, x_2)$. Then

$$\mathbb{A}F_1(x) = 0 \tag{S4.14}$$

for all $x \in \mathbb{N}_0$, which shows that $\mathbb{E}[X_1(t) - X_2(t)] = X_1(0) - X_2(0) = \alpha$ for all $t \geq 0$. Therefore the difference between the first-order moments remains constant over time. However, we now show that the trajectories of $\mathbb{E}[X_1(t)]$ and $\mathbb{E}[X_2(t)]$ grow unboundedly with time.

Let $Z(t) = X_1(t) - X_2(t)$. For any $M > 0$, Markov's inequality and $X_2(t) \geq 0$ imply that

$$\begin{aligned} \mathbb{E}[X_1(t)] &\geq M\mathbb{P}(X_1(t) \geq M) \\ &\geq M\mathbb{P}(X_1(t) \geq M + X_2(t)) \\ &= M\mathbb{P}(Z(t) \geq M) \end{aligned} \tag{S4.15}$$

Note that the process $(Z(t))_{t\geq 0}$ starts at α and it remains unaffected by the third reaction in (S4.8). From the random time-change representation of Kurtz (see Chapter 4 in [6]) it is immediate that the process $(Z(t))_{t\geq 0}$ can be represented as

$$Z(t) = \alpha + Y_1(t) - Y_2(t),$$

where Y_1 and Y_2 are independent, unit rate Poisson processes. Using the property of independent and stationary increments of a Poisson process, and the Central Limit Theorem we can conlcude that for $i = 1, 2$

$$\left(\frac{Y_i(t) - t}{\sqrt{t}} \right)$$

converges in distribution to a standard normal random variable as $t \to \infty$. This fact along with the independence of $Y_1(t)$ and $Y_2(t)$ implies that as $t \to \infty$

$$\frac{Z(t)}{\sqrt{t}} = \frac{\alpha}{\sqrt{t}} + \left(\frac{Y_1(t) - t}{\sqrt{t}} \right) - \left(\frac{Y_2(t) - t}{\sqrt{t}} \right),$$

converges in distribution to a normal random variable with mean 0 and variance 2. Hence

$$\lim_{t\to\infty} \mathbb{P}(Z(t) \geq M) = \lim_{t\to\infty} \mathbb{P}\left(\frac{Z(t)}{\sqrt{t}} \geq \frac{M}{\sqrt{t}} \right) = \frac{1}{2}. \tag{S4.16}$$

Therefore from (S4.15) we obtain

$$\liminf_{t\to\infty} \mathbb{E}[X_1(t)] \geq \frac{M}{2}.$$

Since M is arbitrary, we can conclude that $\mathbb{E}[X_1(t)] \to \infty$ as $t \to \infty$. Furthermore, Jensen's inequality shows that all the moments of $X_1(t)$ grow unboundedly with time. From the symmetry of the reaction network (S4.8) it is immediate that the same conclusions hold for $X_2(t)$.

Perhaps interestingly, the variance of $Z(t)$ has a simple closed-form expression. To see this observe that for the function $F_2(x) := (x_1 - x_2)^2$ we have

$$\begin{aligned} \mathbb{A}F_2(x) &= (x_1 - x_2 + 1)^2 - (x_1 - x_2)^2 + (x_1 - x_2 - 1)^2 - (x_1 - x_2)^2 \\ &= 2. \end{aligned} \tag{S4.17}$$

Therefore Dynkin's theorem implies that $\mathbb{E}[Z(t)^2] = 2t + \alpha^2$ and hence the variance of $Z(t)$ is just $2t$, showing explicitly that it grows unboundedly with time.

Note that unbounded growth of all the moments does not necessarily imply that the process is non-ergodic. However for this example we show that this is indeed the case and the Markov process is

non-ergodic. We argue by contradiction. Suppose that the process is ergodic and let π be the unique stationary distribution. For any $\varepsilon \in (0, 1/2)$ there exists a $M > 0$ large enough such that

$$\pi\left(\{(x_1, x_2) \in \mathbb{N}_0^2 : x_1 < M\}\right) \geq 1 - \varepsilon\,.$$

Since the process is ergodic, then we must have

$$1 - \varepsilon \leq \pi\left(\{(x_1, x_2) \in \mathbb{N}_0^2 : x_1 < M\}\right) = \lim_{t\to\infty} \mathbb{P}(X_1(t) < M) = 1 - \lim_{t\to\infty} \mathbb{P}(X_1(t) \geq M). \tag{S4.18}$$

However, $X_1(t) \geq Z(t)$ for any t and hence using (S4.16) we get

$$\lim_{t\to\infty} \mathbb{P}(X_1(t) \geq M) \geq \lim_{t\to\infty} \mathbb{P}(Z(t) \geq M) = \frac{1}{2}.$$

Substituting this inequality in (S4.18) we obtain $1 - \varepsilon \leq 1/2$ which is a contradiction since $\varepsilon \in (0, 1/2)$. Hence the process is non-ergodic.

S4.4 Details on the attractive compact set calculation

Let us consider the stochastic bimolecular chemical reaction network

$$\begin{array}{rcl} \emptyset & \xrightarrow{k} & \boldsymbol{S_1} \\ \boldsymbol{S_1} & \xrightarrow{\gamma_1} & \emptyset \\ \boldsymbol{S_1} + \boldsymbol{S_1} & \xrightarrow{b} & \boldsymbol{S_2} \\ \boldsymbol{S_2} & \xrightarrow{u} & \boldsymbol{S_1} + \boldsymbol{S_1} \\ \boldsymbol{S_2} & \xrightarrow{\gamma_2} & \emptyset \end{array} \tag{S4.19}$$

which is easily seen to be represented by an irreducible Markov process. In this case, the condition (S1.4a) becomes

$$\begin{aligned} &kv_1 - c_1 + \left(-\gamma_1 v_1 + c_2 v_1 - \frac{b}{2}(v_2 - 2v_1)\right) x_1 + \frac{b}{2}(v_2 - 2v_1)x_1^2 \\ &+ (u(2v_1 - v_2) + c_2 v_2 - \gamma_2 v_2)\, x_2 \leq 0. \end{aligned} \tag{S4.20}$$

Noting that $S_q = \begin{bmatrix} -2 \\ 1 \end{bmatrix}$, and hence setting $v = \begin{bmatrix} 1 \\ 2 \end{bmatrix} \in \mathcal{N}_q$ the above inequality becomes

$$k - c_1 + (-\gamma_1 + c_2)\, x_1 + 2\,(c_2 - \gamma_2)\, x_2 \leq 0,\ \ x \in \mathbb{R}_{\geq 0}^2. \tag{S4.21}$$

Condition (S4.21) is affine in x and therefore easy to verify. Choosing $c_1 = k$ and $c_2 = \min\{\gamma_1, \gamma_2\}$ implies that $\widehat{C}_1 = c_1/c_2$ and that

$$\lim_{t\to\infty} \left(\mathbb{E}[X_1(t)] + 2\mathbb{E}[X_2(t)]\right) \leq \frac{k}{\min\{\gamma_1, \gamma_2\}}. \tag{S4.22}$$

Letting, for instance, $k = 10$, $\gamma_1 = 2$, $b = 10$, $u = 1$ and $\gamma_2 = 2$ as well as $v = \begin{bmatrix} 1 & 2 \end{bmatrix}^T$, we get the optimal cost of 5 meaning that for $X_1(0) = X_2(0) = 0$ we have

$$\lim_{t\to\infty} \mathbb{E}[X_1(t)] + 2\mathbb{E}[X_2(t)] \leq 5. \tag{S4.23}$$

Monte-Carlo simulations yield the values $E_1 = 1.17 \pm 0.01$ and $E_2 = 1.927 \pm 0.02$ for the equilibrium values of the average number of species $\boldsymbol{S_1}$ and $\boldsymbol{S_2}$. Inserting these values in the equation (S4.23) yields

$$E_1 + 2E_2 = 5.024 \pm 0.05 \tag{S4.24}$$

showing that the compact set agrees very well with the equilibrium values (although this is a necessary condition only). As a by-product, this also proves that the stochastic chemical reaction network is ergodic and that all the moments are bounded and globally converging.

S5 Details on the feedback loop example

The case of dimerization is considered first and is later generalized to the multimerization case.

S5.1 Dimerization

Let us consider the feedback network with dimerization

$$\begin{array}{rcl} \emptyset & \xrightarrow{f(\mathbf{S_3})} & \boldsymbol{S_1} \\ \boldsymbol{S_1} & \xrightarrow{k_2} & \boldsymbol{S_1} + \boldsymbol{S_2} \\ \boldsymbol{S_2} + \boldsymbol{S_2} & \rightleftharpoons & \boldsymbol{S_3} \\ \boldsymbol{S_i} & \xrightarrow{\gamma_i} & \emptyset,\ i = 1,2,3. \end{array} \tag{S5.1}$$

where the function $f : \mathbb{R}_{\geq 0} \to \mathbb{R}_{\geq 0}$ is bounded, e.g. an inhibiting Hill function. Above, $\boldsymbol{S_1}$, $\boldsymbol{S_2}$ and $\boldsymbol{S_3}$ are the mRNA, protein and dimer, respectively. Such a network is represented in Fig. 1. We have the following result:

Result S5.1. *For any positive values of the rate parameters and any bounded nonnegative function f, the feedback loop network with dimerization* (S5.1) *is ergodic and all the moments are bounded and globally converging.*

Proof. The matrix S_q is given, in this case, by

$$S_q = \begin{bmatrix} 0 \\ -2 \\ 1 \end{bmatrix} \tag{S5.2}$$

and therefore we have that

$$\mathcal{N}_q = \text{Span}_{>0} \begin{bmatrix} 1 & 0 \\ 0 & 1 \\ 0 & 2 \end{bmatrix} \tag{S5.3}$$

where $\text{Span}_{>0}$ denotes the positive span, i.e. all possible linear combinations of the columns resulting in positive vectors. The left-hand side of Condition **DD** can be upper-bounded by the expression $x^\mathsf{T} Av + b^\mathsf{T} v$ where

$$A = \begin{bmatrix} -\gamma_1 & k_2 & 0 \\ 0 & -\gamma_2 & 0 \\ 0 & 0 & -\gamma_3 \end{bmatrix}, \quad b = \begin{bmatrix} \sup\limits_{y \geq 0}\{f(y)\} \\ 0 \\ 0 \end{bmatrix}. \tag{S5.4}$$

From Proposition S4.1, the ergodicity of the Markov process is ensured if there exist $\theta_1, \theta_2 > 0$ such that

$$\begin{bmatrix} -\gamma_1 & k_2 & 0 \\ 0 & -\gamma_2 & 0 \\ 0 & 0 & -\gamma_3 \end{bmatrix} \begin{bmatrix} \theta_1 \\ \theta_2 \\ 2\theta_2 \end{bmatrix} < 0. \tag{S5.5}$$

This is equivalent to the existence of $\theta_1, \theta_2 > 0$ such that

$$\begin{bmatrix} -\gamma_1 & k_2 \\ 0 & -\gamma_2 \end{bmatrix} \begin{bmatrix} \theta_1 \\ \theta_2 \end{bmatrix} < 0. \tag{S5.6}$$

From positive system theory [8], for all $\gamma_1, \gamma_2 > 0$ and $k_2 > 0$, there exist $\theta_1, \theta_2 > 0$ such that Condition (S5.6) is satisfied since the matrix on the left-hand side is Hurwitz-stable. Therefore, using Proposition S4.1, we can conclude that the feedback loop with dimerization is ergodic. Suitable choice for c_1 and c_2 are given by $c_1 = \theta_1 \cdot \sup_{y \geq 0}\{f(y)\}$ and c_2 can be set arbitrarily close to $\min\limits_i\{\gamma_i\}$ through an appropriate choice for $\theta_1, \theta_2 > 0$. To show that all the moments exist, then it is enough to show that the Condition **DD** holds with $c_5 = 0$. From the fact that $v \in \mathcal{N}_q$ and the boundedness of the function f, we can then conclude that the condition indeed holds with $c_5 = 0$. The conclusion follows. □

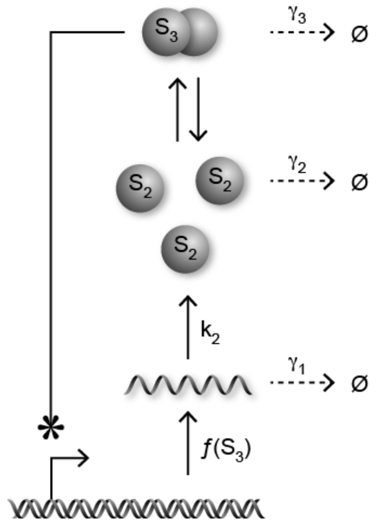

Figure 1: Feedback loop with arbitrary feedback rule.

S5.2 Multimerization with full degradation

Let us consider now the case where a multimerization of order N takes place before acting back on gene expression. In this case, we have the following reaction network

$$\begin{array}{rcl} \emptyset & \xrightarrow{f(\boldsymbol{S_N})} & \boldsymbol{S_0} \\ \boldsymbol{S_0} & \xrightarrow{k_1} & \boldsymbol{S_0} + \boldsymbol{S_1} \\ \boldsymbol{S_1} + \boldsymbol{S_{1+i}} & \rightleftharpoons & \boldsymbol{S_{2+i}},\ i = 0, \dots, N-2 \\ \boldsymbol{S_i} & \xrightarrow{\gamma_i} & \emptyset,\ i = 0, \dots, N. \end{array} \tag{S5.7}$$

involving $N+1$ species and $N-1$ bimolecular reactions. Again the function $f : \mathbb{R}_{\geq 0} \to \mathbb{R}_{\geq 0}$ is bounded on its domain.

We have the following result:

Result S5.2. *For any positive values of the rate parameters and any bounded nonnegative function f, the feedback loop network* (S5.7) *is ergodic and all the moments are bounded and globally converging.*

Proof. The stoichiometry matrix restricted to bimolecular reactions is given by

$$S_q = \begin{bmatrix} S_q^{11} & S_q^{12} \\ S_q^{21} & S_q^{22} \\ 0 & S_q^{32} \end{bmatrix} \tag{S5.8}$$

where $S_q^{11} = \begin{bmatrix} 0 \\ -2 \end{bmatrix}$, $S_q^{12} = \begin{bmatrix} 0_{1\times(N-2)} \\ -\mathbb{1}_{1\times(N-2)} \end{bmatrix}$, $S_q^{21} = \begin{bmatrix} 1 \\ 0_{(N-3)\times 1} \end{bmatrix}$, $S_q^{32} = \begin{bmatrix} 0_{1\times(N-3)} & 1 \end{bmatrix}$ and S_q^{22} is a $(N-2)\times(N-2)$ lower bidiagonal matrix with -1 entries on the main diagonal and 1 entries on the lower one. For this network, we have

$$\mathcal{N}_q = \mathrm{Span}_{>0} \begin{bmatrix} 1 & 0 \\ 0 & 1 \\ 0 & 2 \\ \vdots & \vdots \\ 0 & N \end{bmatrix} \tag{S5.9}$$

and ergodicity holds if there exist $\theta_1, \theta_2 > 0$ such that the inequality

$$\begin{bmatrix} -\gamma_0 & k_1 & 0 & \dots & 0 \\ 0 & -\gamma_1 & 0 & \dots & 0 \\ 0 & 0 & -\gamma_2 & \dots & 0 \\ 0 & 0 & 0 & \ddots & \vdots \\ 0 & 0 & 0 & 0 & -\gamma_N \end{bmatrix} \begin{bmatrix} \theta_1 \\ \theta_2 \\ 2\theta_2 \\ \vdots \\ N\theta_2 \end{bmatrix} < 0 \tag{S5.10}$$

is satisfied. This problem equivalently reduces to

$$\begin{bmatrix} -\gamma_0 & k_1 \\ 0 & -\gamma_1 \end{bmatrix} \begin{bmatrix} \theta_1 \\ \theta_2 \end{bmatrix} < 0 \tag{S5.11}$$

which is obviously feasible from the stability of the matrix and positive system theory. Therefore, we can conclude from Proposition S4.1 and the boundedness of the function f that the system is ergodic and that all the moments are bounded and globally converging. Moreover, c_2 can be made arbitrarily close to $\min_i\{\gamma_i\}$ and c_1 is given by $\theta_1 \cdot \sup_{y \geq 0}\{f(y)\}$. □

S5.3 Multimerization with partial degradation

Let us consider now the case where a multimerization of order N takes place before acting on the gene expression and that multimers degrade in the multimer of lower order:

$$\begin{array}{rcl} \emptyset & \xrightarrow{f(\mathbf{S_N})} & \boldsymbol{S_0} \\ \boldsymbol{S_0} & \xrightarrow{k_1} & \boldsymbol{S_0} + \boldsymbol{S_1} \\ \boldsymbol{S_1} + \boldsymbol{S_{1+i}} & \rightleftharpoons & \boldsymbol{S_{2+i}},\ i = 0, \dots, N-2 \\ \boldsymbol{S_0} & \xrightarrow{\gamma_0} & \emptyset \\ \boldsymbol{S_1} & \xrightarrow{\gamma_1} & \emptyset \\ \boldsymbol{S_i} & \xrightarrow{\rho_i\gamma_1} & \boldsymbol{S_{i-1}},\ i = 2, \dots, N \end{array} \tag{S5.12}$$

where ρ_i's are positive parameters and $f : \mathbb{R}_{\geq 0} \to \mathbb{R}_{\geq 0}$ is again a bounded function on its domain. We then have the following result:

Result S5.3. *For any positive values of the rate parameters and any bounded nonnegative function $f(\cdot)$, the feedback loop* (S5.12) *is ergodic and all the moments are bounded and globally converging.*

Proof. The matrix S_q is the same as in the previous example and ergodicity holds if there exist $\theta_1, \theta_2 > 0$ such that

$$\begin{bmatrix} -\gamma_0 & k_1 & 0 & \dots & 0 \\ 0 & -\gamma_1 & 0 & \dots & 0 \\ 0 & \rho_2\gamma_1 & -\rho_2\gamma_1 & \dots & 0 \\ 0 & 0 & 0 & \ddots & \vdots \\ 0 & 0 & 0 & \rho_N\gamma_1 & -\rho_N\gamma_1 \end{bmatrix} \begin{bmatrix} \theta_1 \\ \theta_2 \\ 2\theta_2 \\ \vdots \\ N\theta_2 \end{bmatrix} < 0. \tag{S5.13}$$

Using the same arguments as in the previous examples, we can conclude from Proposition S4.1 and the boundedness of the function f that the reaction network is ergodic and that all the moments are bounded and globally converging. Moreover, c_2 can be made arbitrarily close to $\min\{\gamma_0, \gamma_1, \rho_2\gamma_1, \dots, \rho_N\gamma_1\}$ and $c_1 = \theta_1 \cdot \sup_{y \geq 0}\{f(y)\}$. □

S6 Details on the stochastic switch example

S6.1 Stochastic switch with direct interaction

Let us consider the stochastic switch of [14] described by following network

$$\begin{array}{rcl} \emptyset & \xrightarrow{f_1(\boldsymbol{S_2^1})} & \boldsymbol{S_1^0} \\ \boldsymbol{S_1^0} & \xrightarrow{k_1} & \boldsymbol{S_1^0}+\boldsymbol{S_1^1} \\ \emptyset & \xrightarrow{f_2(\boldsymbol{S_1^1})} & \boldsymbol{S_2^0} \\ \boldsymbol{S_2^0} & \xrightarrow{k_2} & \boldsymbol{S_2^0}+\boldsymbol{S_2^1} \\ \boldsymbol{S_i^j} & \xrightarrow{\gamma_{i,j}} & \emptyset,\ i,j=1,2. \end{array} \tag{S6.1}$$

where the functions f_1 and f_2 are inhibiting Hill functions (but can be generalized to any bounded functions). Above, for each gene i, the species $\boldsymbol{S_i^0}$ and $\boldsymbol{S_i^1}$ represent mRNAs and proteins, respectively. We then have the following result:

Result S6.1. *For any values of the rate parameters and any bounded functions $f_1(\cdot)$ and $f_2(\cdot)$, the stochastic switch* (S6.1) *is ergodic and all the moments are bounded and globally converging.*

Proof. First note that for the network (S6.1), we have that

$$\sum_{k=1}^{6}\lambda_k(x)\langle v,\zeta_k\rangle \le x^{\mathsf{T}}Av+b,\ x\in\mathbb{N}_0^4 \tag{S6.2}$$

where

$$A=\begin{bmatrix} -\gamma_{1,0} & k_1 & 0 & 0 \\ 0 & -\gamma_{1,1} & 0 & 0 \\ 0 & 0 & -\gamma_{2,0} & k_2 \\ 0 & 0 & 0 & -\gamma_{2,1} \end{bmatrix},\ b=\begin{bmatrix} \sup\limits_{y\ge0} f_1(y) \\ 0 \\ \sup\limits_{y\ge0} f_2(y) \\ 0 \end{bmatrix}. \tag{S6.3}$$

Since the matrix A is Hurwitz-stable and Metzler, then for all possible rate parameters, there exists a $v>0$ such that $Av<0$. From Proposition S3.1, we can then conclude that the stochastic reaction network is ergodic. Moreover, c_2 can be set arbitrarily close to $\min\limits_{i,j}\{\gamma_{i,j}\}$ and $c_1=v_1\sup_{y\ge0}f_1(y)+v_3\sup_{y\ge0}f_2(y)$. To show that all the moments exist, we need to prove that Condition **DD** holds with $c_5=0$. Noting then that the functions f_1 and f_2 are bounded from above, we can bound the left-hand side of (S1.4b) by an affine function of x, showing therefore that c_5 can be set to 0. The proof is complete. □

S6.2 Stochastic switch with interaction by multimerization

Consider now a switch whose genes interact through multimers of their respective proteins as

$$\begin{array}{rcl} \emptyset & \xrightarrow{f_1(\boldsymbol{S_2^{N_2}})} & \boldsymbol{S_1^0} \\ \boldsymbol{S_1^0} & \xrightarrow{k_1^1} & \boldsymbol{S_1^0}+\boldsymbol{S_1^1} \\ \boldsymbol{S_1^1}+\boldsymbol{S_1^i} & \xrightarrow{b_1^i} & \boldsymbol{S_1^{i+1}},\ i=1,\ldots,N_1-1 \\ \emptyset & \xrightarrow{f_2(\boldsymbol{S_1^{N_1}})} & \boldsymbol{S_2^0} \\ \boldsymbol{S_2^0} & \xrightarrow{k_2} & \boldsymbol{S_2^0}+\boldsymbol{S_2^1} \\ \boldsymbol{S_2^1}+\boldsymbol{S_2^i} & \xrightarrow{b_2^i} & \boldsymbol{S_2^{i+1}},\ i=1,\ldots,N_2-1 \\ \boldsymbol{S_1^j} & \xrightarrow{\gamma_{i,j}} & \emptyset,\ j=1,\ldots,N_1 \\ \boldsymbol{S_1^2} & \xrightarrow{\gamma_{i,j}} & \emptyset,\ j=1,\ldots,N_2 \end{array} \tag{S6.4}$$

where N_1 and N_2 are the multimerization orders of the proteins of gene 1 and 2, respectively. This network involves N_1+N_2-2 bimolecular reactions while the number of different species is N_1+N_2+2. The functions f_1 and f_2 are again inhibiting Hill functions. We then have the following result:

Result S6.2. *For any values of the rate parameters and any bounded functions $f_1(\cdot)$ and $f_2(\cdot)$, the reaction network* (S6.4) *is ergodic and all the moments are bounded and globally converging.*

Proof. The matrix S_q is given by

$$S_q = \begin{bmatrix} S_q^1 & 0 \\ 0 & S_q^2 \end{bmatrix} \tag{S6.5}$$

where

$$S_q^i = \begin{bmatrix} M_{11}^i & M_{12}^i \\ M_{21}^i & M_{22}^i \\ 0 & M_{32}^i \end{bmatrix} \tag{S6.6}$$

where $M_{11}^i = \begin{bmatrix} 0 \\ -2 \end{bmatrix}$, $M_{12}^i = \begin{bmatrix} 0_{1\times(N_i-2)} \\ -\mathbb{1}_{1\times(N_i-2)} \end{bmatrix}$, $M_{21}^i = \begin{bmatrix} 1 \\ 0_{(N_i-3)\times 1} \end{bmatrix}$, $M_{32}^i = \begin{bmatrix} 0_{1\times(N_i-3)} & 1 \end{bmatrix}$ and M_{22}^i is a $(N_i-2)\times(N_i-2)$ lower bidiagonal matrix with -1 entries on the main diagonal and 1 entries on the lower one.

Therefore, the set $\mathcal{N}_q$ is given in this case by

$$\mathcal{N}_q = \mathrm{Span}_{>0} \begin{bmatrix} \mathcal{N}^1 & 0 \\ 0 & \mathcal{N}^2 \end{bmatrix} \tag{S6.7}$$

where

$$\mathcal{N}^i := \begin{bmatrix} 1 & 0 \\ 0 & 1 \\ 0 & 2 \\ \vdots & \vdots \\ 0 & N_i \end{bmatrix}. \tag{S6.8}$$

Thus, the network is ergodic if there exist $\theta_i > 0$, $i = 1, \ldots, 4$ such that

$$\begin{bmatrix} -\gamma_1^0 & k_1^2 & 0 & \ldots & 0 \\ 0 & -\gamma_1^1 & 0 & \ldots & 0 \\ 0 & 0 & -\gamma_1^2 & \ldots & 0 \\ 0 & 0 & 0 & \ddots & \vdots \\ 0 & 0 & 0 & 0 & -\gamma_1^{N_1} \end{bmatrix} \begin{bmatrix} \theta_1 \\ \theta_2 \\ 2\theta_2 \\ \vdots \\ N_1\theta_2 \end{bmatrix} < 0 \tag{S6.9}$$

and

$$\begin{bmatrix} -\gamma_2^0 & k_1^1 & 0 & \ldots & 0 \\ 0 & -\gamma_2^1 & 0 & \ldots & 0 \\ 0 & 0 & -\gamma_2^2 & \ldots & 0 \\ 0 & 0 & 0 & \ddots & \vdots \\ 0 & 0 & 0 & 0 & -\gamma_2^{N_2} \end{bmatrix} \begin{bmatrix} \theta_3 \\ \theta_4 \\ 2\theta_4 \\ \vdots \\ N_1\theta_4 \end{bmatrix} < 0 \tag{S6.10}$$

hold. Similar to the previous examples, we can conclude from Proposition S4.1 and the boundedness of the functions f_i's that, for all positive values for the reaction rates, the reaction network is ergodic and that all the moments are bounded and globally converging. Moreover, c_2 can be made arbitrarily close to $\min\limits_{i,j}\{\gamma_i^j\}$ and $c_1 = \theta_1 \sup_{y\ge 0}\{f_1(y)\} + \theta_3 \sup_{y\ge 0}\{f_2(y)\}$. □

S7 Details on the repressilator example

Let us consider the following N-gene repressilator [5] (see also Fig. 2):

$$\begin{array}{lcl}
\emptyset & \xrightarrow{f_1(\boldsymbol{S_N^1})} & \boldsymbol{S_1^1} \\
\emptyset & \xrightarrow{f_2(\boldsymbol{S_1^1})} & \boldsymbol{S_2^1} \\
\emptyset & \xrightarrow{f_3(\boldsymbol{S_2^1})} & \boldsymbol{S_3^1} \\
\vdots & \vdots & \vdots \\
\emptyset & \xrightarrow{f_N(\boldsymbol{S_{N-1}^1})} & \boldsymbol{S_N^1} \\
\boldsymbol{S_1^1} & \xrightarrow{k_1} & \boldsymbol{S_1^1} + \boldsymbol{S_1^2} \\
\boldsymbol{S_2^1} & \xrightarrow{k_2} & \boldsymbol{S_2^1} + \boldsymbol{S_2^2} \\
\boldsymbol{S_3^1} & \xrightarrow{k_3} & \boldsymbol{S_3^1} + \boldsymbol{S_3^2} \\
\vdots & \vdots & \vdots \\
\boldsymbol{S_N^1} & \xrightarrow{k_n} & \boldsymbol{S_N^1} + \boldsymbol{S_N^2} \\
\boldsymbol{S_i^1} & \xrightarrow{\gamma_i} & \emptyset,\ i = 1, \ldots, N \\
\boldsymbol{S_i^2} & \xrightarrow{\delta_i} & \emptyset,\ i = 1, \ldots, N
\end{array} \tag{S7.1}$$

where $f_i(x) = \alpha_i + \beta_i/(1+x^n)$, $\alpha_i, \beta_i, n > 0$. Above, $\boldsymbol{S_i^1}$ and $\boldsymbol{S_i^2}$ are the mRNA and protein corresponding to gene i. We then have the following result:

Result S7.1. *For all positive values of the parameters, the reaction network* (S7.1) *is ergodic and all the moments are bounded and globally converging.*

Proof. Similar to the previous example, we can use an upper-bound on the left-hand side of Condition **DD**. Then, from Proposition S3.1 and the boundedness of the functions f_i's, we can conclude that the network is ergodic and that all the moments are bounded and globally converging. Moreover, c_2 can be made arbitrarily close to $\min_i\{\gamma_i, \delta_i\}$ and $c_1 = \sum_{i=1}^N (\alpha_i + \beta_i)v_i$. □

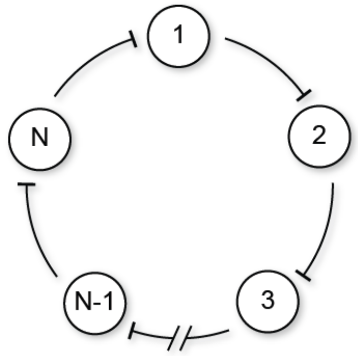

Figure 2: N-gene repressilator.

It is important to stress that multimerization with full or partial degradation can be easily incorporated in the model in the same way as for the feedback loop or the stochastic switch. It is therefore possible to state that the N-gene repressilator with an arbitrary degree of multimerization is ergodic and all the moments are bounded and globally converging.

S8 Details on the SIR model example

Let us consider the following open stochastic SIR-model

$$\begin{array}{rcl} \emptyset & \xrightarrow{k_s} & \boldsymbol{S} \\ \emptyset & \xrightarrow{k_i} & \boldsymbol{I} \\ \boldsymbol{S} & \xrightarrow{\gamma_s} & \emptyset \\ \boldsymbol{I} & \xrightarrow{\gamma_i} & \emptyset \\ \boldsymbol{R} & \xrightarrow{\gamma_r} & \emptyset \\ \boldsymbol{S}+\boldsymbol{I} & \longrightarrow & 2\boldsymbol{I} \\ \boldsymbol{I} & \xrightarrow{k_{ir}} & \boldsymbol{R} \\ \boldsymbol{R} & \xrightarrow{k_{rs}} & \boldsymbol{S}. \end{array} \tag{S8.1}$$

where $\boldsymbol{S}$, $\boldsymbol{I}$ and $\boldsymbol{R}$ represent the population of susceptible, infectious and recovered people. Birth and death reactions represent people entering and leaving the process, respectively. The only bimolecular reaction is contamination which transforms one susceptible person into an infectious one. The two last reactions represent infectious people recovering and, then, becoming susceptible again.

We then have the following result:

Result S8.1. *For all positive values of the rate parameters, the stochastic SIR-model* (S8.1) *is ergodic and that all the moments are bounded and globally converging.*

Proof. The restriction of the stoichiometric matrix to bimolecular reactions is given by

$$S_q = \begin{bmatrix} -1 \\ 1 \\ 0 \end{bmatrix} \tag{S8.2}$$

and hence we have that

$$\mathcal{N}_q = \text{Span}_{>0} \begin{bmatrix} 1 & 0 \\ 1 & 0 \\ 0 & 1 \end{bmatrix}. \tag{S8.3}$$

The matrices A and b defined in (S4.1) for this network are given by

$$A = \begin{bmatrix} -\gamma_s & 0 & 0 \\ 0 & -\gamma_i - k_{ir} & k_{ir} \\ k_{rs} & 0 & -\gamma_r - k_{rs} \end{bmatrix}, \ b = \begin{bmatrix} k_s \\ k_i \\ 0 \end{bmatrix}. \tag{S8.4}$$

Using Proposition S4.1 we can conclude that the network is ergodic and all the moments are bounded and globally converging if there exist $\theta_1, \theta_2 > 0$ such that

$$\begin{bmatrix} -\gamma_s & 0 & 0 \\ 0 & -\gamma_i - k_{ir} & k_{ir} \\ k_{rs} & 0 & -\gamma_r - k_{rs} \end{bmatrix} \begin{bmatrix} \theta_1 \\ \theta_1 \\ \theta_2 \end{bmatrix} < 0. \tag{S8.5}$$

This can be equivalently reformulated as

$$\begin{bmatrix} -\gamma_i - k_{ir} & k_{ir} \\ k_{rs} & -\gamma_r - k_{rs} \end{bmatrix} \begin{bmatrix} \theta_1 \\ \theta_2 \end{bmatrix} < 0. \tag{S8.6}$$

Since the matrix in the above inequality is Hurwitz-stable for all positive values of the parameters, there exist $\theta_1, \theta_2 > 0$ such that the inequality holds. Moreover, c_2 can be made arbitrarily close to the additive inverse of the Frobenius eigenvalue of the matrix in (S8.5) and $c_1 = (k_s + k_i)\theta_1$. Therefore we can conclude from Proposition S4.1 that the SIR-model (S8.1) is structurally ergodic and that all the moments are bounded and globally converging. □

S9 Details on the circadian clock example

We consider here a more complex example, the circadian clock model of [15] described by the following set of reactions:

$$
\begin{array}{llrcl}
R_1 & : & \boldsymbol{S_1} + \boldsymbol{S_4} & \xrightarrow{\gamma_A} & \boldsymbol{S_2}\\
R_2 & : & \boldsymbol{S_2} & \xrightarrow{\theta_A} & \boldsymbol{S_1} + \boldsymbol{S_4}\\
R_3 & : & \boldsymbol{S_5} + \boldsymbol{S_4} & \xrightarrow{\gamma_R} & \boldsymbol{S_6}\\
R_4 & : & \boldsymbol{S_6} & \xrightarrow{\theta_R} & \boldsymbol{S_5} + \boldsymbol{S_4}\\
R_5 & : & \boldsymbol{S_2} & \xrightarrow{\alpha'_A} & \boldsymbol{S_2} + \boldsymbol{S_3}\\
R_6 & : & \boldsymbol{S_1} & \xrightarrow{\alpha_A} & \boldsymbol{S_1} + \boldsymbol{S_3}\\
R_7 & : & \boldsymbol{S_3} & \xrightarrow{\delta_{M_A}} & \emptyset\\
R_8 & : & \boldsymbol{S_3} & \xrightarrow{\beta_A} & \boldsymbol{S_3} + \boldsymbol{S_4}\\
R_9 & : & \boldsymbol{S_4} & \xrightarrow{\delta_A} & \emptyset\\
R_{10} & : & \boldsymbol{S_6} & \xrightarrow{\alpha'_R} & \boldsymbol{S_6} + \boldsymbol{S_7}\\
R_{11} & : & \boldsymbol{S_5} & \xrightarrow{\alpha_R} & \boldsymbol{S_5} + \boldsymbol{S_7}\\
R_{12} & : & \boldsymbol{S_7} & \xrightarrow{\delta_{M_R}} & \emptyset\\
R_{13} & : & \boldsymbol{S_7} & \xrightarrow{\beta_R} & \boldsymbol{S_7} + \boldsymbol{S_8}\\
R_{14} & : & \boldsymbol{S_8} & \xrightarrow{\delta_R} & \emptyset\\
R_{15} & : & \boldsymbol{S_4} + \boldsymbol{S_8} & \xrightarrow{\gamma_C} & \boldsymbol{S_9}\\
R_{16} & : & \boldsymbol{S_9} & \xrightarrow{\delta_A} & \boldsymbol{S_8}
\end{array}
$$

where $\boldsymbol{S_1} = D_A$, $\boldsymbol{S_2} = D'_A$, $\boldsymbol{S_3} = M_A$, $\boldsymbol{S_4} = A$, $\boldsymbol{S_5} = D_R$, $\boldsymbol{S_6} = D'_R$, $\boldsymbol{S_7} = M_R$, $\boldsymbol{S_8} = R$ and $\boldsymbol{S_9} = C$, according to the notations of [15]; see also Fig. 3. The above network, moreover, admits the conservation of mass relations $X_1(t) + X_2(t) = 1$ and $X_5(t) + X_6(t) = 1$ where $X_i(t)$ denotes the random variable associated with species $\boldsymbol{S_i}$. Using, for instance, the numerical values of [15], we obtain the oscillatory trajectories depicted in Fig. 4.

We then have the following result:

Result S9.1. *For all positive values of the rate parameters, the circadian-clock model is ergodic and all the moments are bounded and converging.*

Proof. The circadian model contains bimolecular reactions corresponding to gene activation (R_1 and R_3) and protein binding (R_{15}). This system contains some species in finite number, i.e. $\boldsymbol{S_1}, \boldsymbol{S_2}, \boldsymbol{S_5}, \boldsymbol{S_6}$, which correspond to genes, and potentially infinite ones corresponding to mRNA and proteins. Let $f(x, v)$ be given by

$$f(x, v) := \sum_{i=1}^{16} \lambda_k(x)\langle v, \zeta_k\rangle. \tag{S9.1}$$

This yields

$$\begin{array}{rcl} f(x,v) &=& \gamma_A x_1 x_4(v_2 - v_1 - v_4) + \theta_A x_2(v_4 + v_1 - v_2) + \gamma_R x_5 x_4(v_6 - v_4 - v_5)\\ && +\ \theta_R x_6(v_4 + v_5 - v_6) + \alpha'_A x_2 v_3 + \alpha_A x_1 v_3 - \delta_{M_A} x_3 v_3 - \delta_A x_4 v_4\\ && +\ \beta_A x_3 v_4 + \alpha'_R x_6 v_7 + \alpha_R x_5 v_7 - \delta_{M_R} x_7 v_7 + \beta_R x_7 v_8\\ && +\ \gamma_C x_4 x_8(v_9 - v_4 - v_8) + \delta_A x_9(v_8 - v_9) - \delta_R x_8 v_8. \end{array} \tag{S9.2}$$

Gathering the terms that are bounded into $\bar{b}(v, x)$ we obtain

$$\begin{array}{rcl} f(x,v) &=& \bar{b}(x,v) + x_3(-\delta_{M_A} v_3 + \beta_A v_4) - \delta_A x_4 v_4 + \gamma_C x_4 x_8(v_9 - v_4 - v_8)\\ && +\ x_7\left[-\delta_{M_R} v_7 + \beta_R v_8\right] - \delta_R v_8 x_8 + \delta_A(v_8 - v_9)x_9\\ && +\ \gamma_A x_1 x_4(v_2 - v_1 - v_4) + \gamma_R x_4 x_5(v_6 - v_5 - v_4)\\ \bar{b}(x,v) &=& \theta_A(v_4 + v_1 - v_2) + \theta_R(v_4 + v_5 - v_6) + \alpha'_A v_3 + \alpha'_R v_7\\ && +\ x_1\left[-\theta_A(v_4 + v_1 - v_2) - \alpha'_A v_3 + \alpha_A v_3\right]\\ && +\ x_5\left[-\theta_R(v_4 + v_5 - v_6) - \alpha'_R v_7 + \alpha_R v_7\right] \end{array} \tag{S9.3}$$

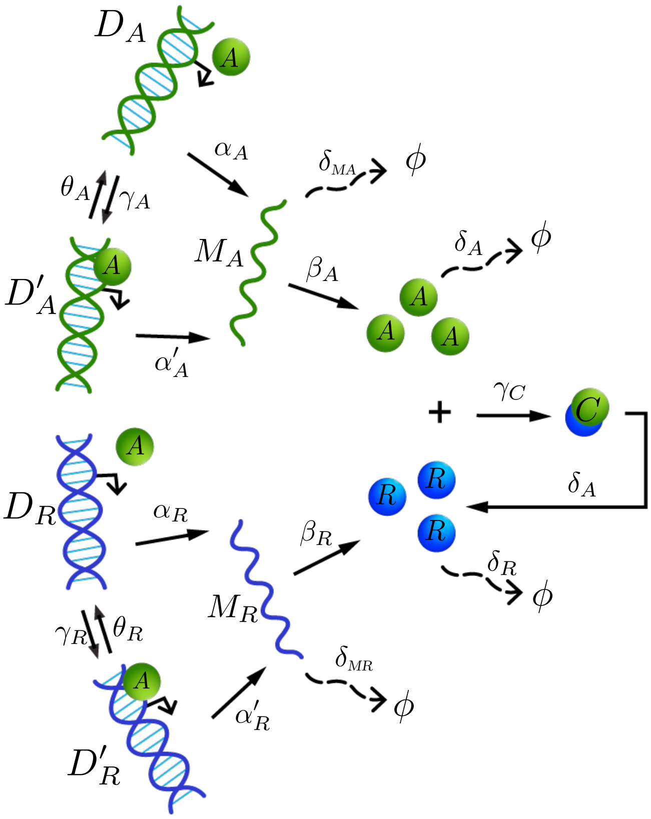

Figure 3: Circadian clock model of [15].

where we have used the change of variables $x_2 = 1 - x_1$ and $x_6 = 1 - x_5$. Since the term $\bar{b}(x, v)$ is bounded, then the only bimolecular reaction we have to take care of is reaction R_{15} since this is the only one that may lead to unbounded trajectories and unbounded moments. The corresponding stoichiometry vector is given by

$$S_q = \begin{bmatrix} 0 \\ 0 \\ 0 \\ -1 \\ 0 \\ 0 \\ 0 \\ -1 \\ 1 \end{bmatrix}. \tag{S9.4}$$

Then, we have

$$\mathcal{N}_q = \mathrm{Span}_{>0} \begin{bmatrix} 1 & 0 & 0 & 0 & 0 & 0 & 0 & 0 \\ 0 & 1 & 0 & 0 & 0 & 0 & 0 & 0 \\ 0 & 0 & 1 & 0 & 0 & 0 & 0 & 0 \\ 0 & 0 & 0 & 1 & 0 & 0 & 0 & 0 \\ 0 & 0 & 0 & 0 & 1 & 0 & 0 & 0 \\ 0 & 0 & 0 & 0 & 0 & 1 & 0 & 0 \\ 0 & 0 & 0 & 0 & 0 & 0 & 1 & 0 \\ 0 & 0 & 0 & 0 & 0 & 0 & 0 & 1 \\ 0 & 0 & 0 & 1 & 0 & 0 & 0 & 1 \end{bmatrix}. \tag{S9.5}$$

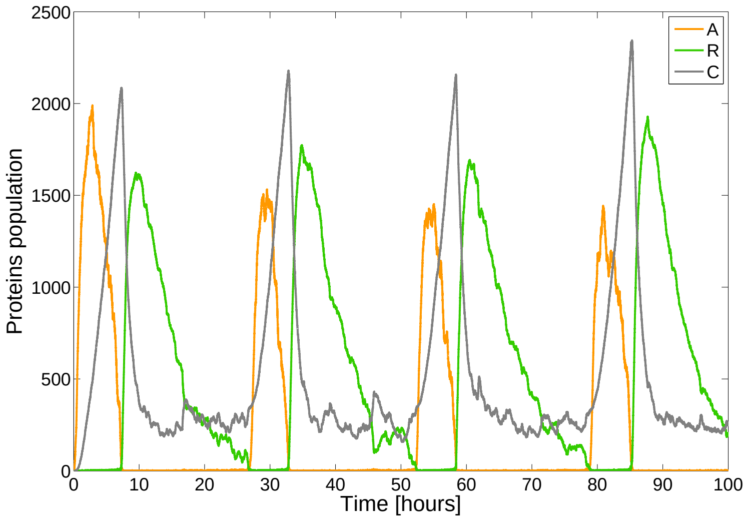

Figure 4: Sample-path of the species of the circadian clock model.

From (S9.3) and Proposition S4.1, ergodicity holds if the inequality

$$\begin{bmatrix} -\delta_{M_A}+c_2 & \beta_A & 0 & 0 & 0 \\ 0 & -\delta_A+c_2 & 0 & 0 & 0 \\ 0 & 0 & -\delta_{M_R}+c_2 & \beta_R & 0 \\ 0 & 0 & 0 & -\delta_R+c_2 & 0 \\ 0 & 0 & 0 & \delta_A & -\delta_A+c_2 \end{bmatrix} \begin{bmatrix} v_3 \\ v_4 \\ v_7 \\ v_8 \\ v_9 \end{bmatrix} \le 0 \tag{S9.6}$$

holds for some $v \in \mathcal{N}_q$ and $c_2 > 0$. From (S9.5), we have

$$\begin{bmatrix} v_3 \\ v_4 \\ v_7 \\ v_8 \\ v_9 \end{bmatrix} = \begin{bmatrix} \theta_3 \\ \theta_4 \\ \theta_7 \\ \theta_8 \\ \theta_4+\theta_8 \end{bmatrix}. \tag{S9.7}$$

It is clear that the matrix on the left-hand side of (S9.6) is Hurwitz-stable and Metzler when $c_2 = 0$. Moreover, expanding the last inequality yields the condition $0 < c_2 \le \delta_A\theta_4/(\theta_4+\theta_8)$. Condition (S9.6) then reduces to

$$\begin{bmatrix} -\delta_{M_A}+c_2 & \beta_A & 0 & 0 \\ 0 & -\delta_A+c_2 & 0 & 0 \\ 0 & 0 & -\delta_{M_R}+c_2 & \beta_R \\ 0 & 0 & 0 & -\delta_R+c_2 \end{bmatrix} \begin{bmatrix} \theta_3 \\ \theta_4 \\ \theta_7 \\ \theta_8 \end{bmatrix} \le 0 \tag{S9.8}$$

which is obviously feasible from the fact that the matrix is Metzler, Hurwitz-stable when $c_2 = 0$ and the fact that the θ_i's are independent. Therefore, there exists $c_2 > 0$ and $\theta_3, \theta_4, \theta_7, \theta_8 > 0$ such that the above inequality holds. Moreover, Condition **DD** holds with the same c_2 and

$$c_1 = \sup_{(x_1,x_5)\in\{0,1\}^2} \{\bar{b}(x,v)\}.$$

We can therefore conclude, from Proposition S4.1, that the circadian clock model of [15] is ergodic and that all the moments are bounded and globally converging. □

S10 Details on the analysis of the p53 model

Let us consider the following stochastic model for p53, see [7]:

$$\begin{array}{rcl}\emptyset & \xrightarrow{k_1} & \boldsymbol{S_1}\\ \boldsymbol{S_1} & \xrightarrow{k_2} & \emptyset\\ \boldsymbol{S_1} & \xrightarrow{f(\boldsymbol{S_1},\boldsymbol{S_3})} & \emptyset\\ \boldsymbol{S_1} & \xrightarrow{k_4} & \boldsymbol{S_1}+\boldsymbol{S_2}\\ \boldsymbol{S_2} & \xrightarrow{k_5} & \boldsymbol{S_3}\\ \boldsymbol{S_3} & \xrightarrow{k_6} & \emptyset.\end{array} \tag{S10.1}$$

where $\mathbf{S_1}$ is the p53 protein, $\mathbf{S_2}$ is the Mdm2 precursor and $\mathbf{S_3}$ is the Mdm2 protein. The rate function $f(x,y) = \frac{k_3 y}{x+k_7}$ implements a nonlinear feedback on the degradation rate of p53. This example shows that non mass-action kinetics can also be considered using the proposed approach.

We then have the following result:

Result S10.1. *For any values of the rate parameters, the oscillatory p53 model* (S10.1) *is ergodic and all the moments are bounded and globally converging.*

Proof. For this model, we indeed have that

$$\begin{array}{rcl}\sum_{k=1}^{6}\lambda_k(x)\langle v,\zeta_k\rangle & \leq & k_1v_1 - k_2x_1v_1 + k_4x_1v_2 + k_5x_2(v_3-v_2) - k_6x_3v_3\\ & = & x^T\begin{bmatrix}-k_2 & k_4 & 0\\ 0 & -k_5 & k_5\\ 0 & 0 & -k_6\end{bmatrix}v + \begin{bmatrix}k_1\\0\\0\end{bmatrix}^T v\end{array} \tag{S10.2}$$

It is clear that the above matrix is Hurwitz-stable and thus that there exists a positive vector $v > 0$ such that $Av < 0$ holds. Hence, we can conclude from Proposition S3.1 that the system is exponentially ergodic. To show that all the moments exist, we need to prove that Condition **DD** holds with $c_5 = 0$. Noting that $\dfrac{k_3xy}{x+k_7} \leq k_3y$ for all $x, y \geq 0$, then the left-hand side of (S1.4b) can be bounded from above by an affine polynomial in x_1, x_2 and x_3, showing therefore that c_5 can be set to 0. The proof is complete. □

S11 Details on the analysis of the Lotka-Volterra model

The key idea in this section to obtain a similar result as in [4], but in a stochastic setting. We consider here a stochastic analogue of the deterministic model

$$\dot{n}_i(t) = \left(r_i - \sum_j b_{ij}n_j(t)\right)n_i(t) \tag{S11.1}$$

where n_i is the population of the ith species and r_i, b_{ij} are model parameters representing birth and competition among species. However, the stochastic analogue of the above deterministic model does not behave nicely since species may go extinct (ergodicity may also not hold). Moreover, the proposed framework is not really devoted to the analysis of closed population models since, for these models, ergodicity can easily be checked from the generator of the Markov chain, which is a finite-dimensional matrix. To avoid this issue, we consider the open stochastic Lotka-Volterra model given by the following reaction network.

$$\begin{array}{rcl}\emptyset & \xrightarrow{\alpha_i} & \boldsymbol{S_i}\\ \boldsymbol{S_i} & \xrightarrow{\beta_i} & \boldsymbol{S_i}+\boldsymbol{S_i}\\ \boldsymbol{S_i}+\boldsymbol{S_j} & \xrightarrow{\gamma_{ij}} & \boldsymbol{S_j}\\ \boldsymbol{S_i} & \xrightarrow{\delta_i} & \emptyset\end{array} \tag{S11.2}$$

where $i = 1, \ldots, N$. The first set of reactions represent immigration/pure birth, the second one is reproduction, the third one is competition due to overpopulation and the last one are deaths/migrations. The difference with the direct stochastic analogue of (S11.1) lies in the presence of immigration and pure death or migration reactions.

We then have the following result which is a stochastic analogue of [4].

Theorem S11.1. *Let $\Gamma(v) = [v_i \gamma_{ij}]$ and assume that one of the following conditions hold:*

1. *there exists $v > 0$ such that the $\Gamma(v)^{\mathsf{T}} + \Gamma(v)$ is copositive and $\beta_i - \delta_i < 0$ for all $i = 1, \ldots, N$;*
2. *there exists $v > 0$ such that the matrix $\Gamma(v)^{\mathsf{T}} + \Gamma(v)$ is positive definite.*

Then the stochastic reaction network (S11.2) *is ergodic and all the moments up to order $\lfloor 1 + \frac{c_2}{c_5} \rfloor - 2$ are bounded and globally converging.*

Proof. Let us first define the following quantities

$$\begin{array}{rcl} M(v) & = & -\frac{1}{2}(\Gamma(v)^{\mathsf{T}} + \Gamma(v)) \\ A & = & \operatorname{diag}_i((\beta_i - \delta_i)v_i) \\ b & = & \begin{bmatrix}\alpha_1 & \ldots & \alpha_n\end{bmatrix}^{\mathsf{T}}. \end{array} \tag{S11.3}$$

The condition (S1.4a) can be rewritten as

$$b^{\mathsf{T}}v + x^{\mathsf{T}}Av + x^{\mathsf{T}}M(v)x \quad \leq \quad c_1 - c_2\langle v, x\rangle. \tag{S11.4}$$

Case 1: Assume that $\delta_i - \beta_i < 0$ and $-M(v)$ is copositive, then c_1 and c_2 can be chosen as $c_1 = \sum_{i=1}^n \alpha_i v_i$ and c_2 can be set arbitrarily close to $\min_i\{\delta_i - \beta_i\}$. Ergodicity then follows.

Case 2: This follows from Proposition S4.5. □

S12 Details on the analysis of the Schlögl model

Let us consider the Schlögl model [13]:

$$\begin{array}{ccccc} \mathbf{A} + 2\mathbf{S} & \xrightarrow{k_1} & 3\mathbf{S} & \xrightarrow{k_2} & \mathbf{A} + 2\mathbf{S} \\ \mathbf{B} & \xrightarrow{k_3} & \mathbf{S} & \xrightarrow{k_4} & \mathbf{B}. \end{array} \tag{S12.1}$$

Let X_A, X_B and X_S denote the number of molecules of species A, B and S respectively. We can see that this model is closed since the quantity $X_A + X_B + X_S$ is preserved over time. Therefore, the state-space is finite since we have $X_A(t) + X_B(t) + X_S(t) = X_A(0) + X_B(0) + X_S(0) < \infty$ for all $t \geq 0$. When the populations of the species A and B are very large in number, then we can assume that they are constant over time (as it is often the case in the literature) and the network (S12.1) becomes

$$\begin{array}{ccccc} 2\mathbf{S} & \xrightarrow{k_1 X_A} & 3\mathbf{S} & \xrightarrow{k_2} & 2\mathbf{S} \\ \emptyset & \xrightarrow{k_3 X_B} & \mathbf{S} & \xrightarrow{k_4} & \emptyset. \end{array} \tag{S12.2}$$

Note that in the form presented above, the network is now open and has state-space $\mathbb{N}_0$, which is infinite. The network also involves a single trimolecular reaction. We have the following result:

Theorem S12.1. *For any positive value of the rate parameters k_1, k_2, k_3, k_4 and any positive value for X_A and X_B, the Schlögl model* (S12.2) *is exponentially ergodic.*

Proof. The drift-condition **DD1** with $V(x) = x$ is given by

$$\frac{k_1 X_A}{2}x(x-1) - \frac{k_2}{6}x(x-1)(x-2) + k_3 X_B - k_4 x \leq c_1 - c_2 x, \ \forall x \in \mathbb{N}_0^d. \tag{S12.3}$$

Let us denote the left-hand side by $p(x)$ and rewrite it as

$$p(x) = -\frac{k_2}{6}x^3 + x^2\frac{k_1X_A + k_2}{2} - x\left(k_4 + \frac{k_2}{3} + \frac{k_1X_A}{2}\right) + k_3X_B. \tag{S12.4}$$

Now pick a $c_2 > 0$ and observe that the condition becomes

$$p(x) + c_2x \leq c_1, \forall x \in \mathbb{N}_0. \tag{S12.5}$$

Since the term of higher degree is negative, then clearly the function $p(x) + c_2x$ admits an upper bound over $x \in \mathbb{N}_0$. Picking then $c_1 \geq 0$ such that

$$c_1 = \sup_{x\geq 0}\{p(x) + c_2x\} \geq \max_{x\in\mathbb{N}_0}\{p(x) + c_2x\} \tag{S12.6}$$

show that for any $c_2 > 0$, we can find a $c_1 \geq 0$ such the drift-condition **DD1** holds.

Finally observe that due to the presence of the birth and death reactions, the state-space of the system is irreducible. Hence the result follows. □

References

[1] A. Berman and R. J. Plemmons. *Nonnegative matrices in the mathematical sciences.* SIAM, Philadelphia, USA, 1994.

[2] S. Boyd and L. Vandenberghe. *Convex Optimization.* Cambridge University Press, Cambridge, MA, USA, 2004.

[3] C. Briat. Robust stability and stabilization of uncertain linear positive systems via integral linear constraints - L_1- and L_∞-gains characterizations. *International Journal of Robust and Nonlinear Control*, 23(17):1932–1954, 2013.

[4] N. Champagnat, P.-E. Jabin, and G. Raoul. Convergence to equilibrium in competitive Lotka-Volterra and chemostat systems. *Compte Rendus de l'Académie des Sciences - Mathématique*, 348(23-24):1267–1272, 2010.

[5] M. B. Elowitz and S. Leibler. A synthetic oscillatory network of transcriptional regulators. *Nature*, 403:335–338, 2000.

[6] S. N. Ethier and T. G. Kurtz. *Markov processes : Characterization and Convergence.* Wiley Series in Probability and Mathematical Statistics: Probability and Mathematical Statistics. John Wiley & Sons Inc., New York, 1986.

[7] N. Geva-Zatorsky, N. Rosenfeld, S. Itzkovitz, R. Milo, A. Sigal, E. Dekel, T. Yarnitzky, Y. Liron, P. Polak, G. Lahav, and U. Alon. Oscillations and variability in the p53 system. *Molecular Systems Biology*, 2:2006.0033, 2006.

[8] W. M. Haddad and V. Chellaboina. Stability theory for nonnegative and compartmental dynamical systems with time delay. *Systems & Control Letters*, 51(5):355–361, 2004.

[9] O. Kallenberg. *Foundations of modern probability.* Probability and its Applications (New York). Springer-Verlag, New York, second edition, 2002.

[10] S. Meyn and R. L. Tweedie. *Markov chains and stochastic stability.* Cambridge University Press, Cambridge, second edition, 2009. With a prologue by Peter W. Glynn.

[11] S. P. Meyn and R. L. Tweedie. Stability of Markovian processes. III. Foster-Lyapunov criteria for continuous-time processes. *Adv. in Appl. Probab.*, 25(3):518–548, 1993.

[12] K. G. Murty and S. N. Kabadi. Some NP-complete problems in quadratic and nonlinear programming. *Mathematical Programming*, 39:117–129, 1987.

[13] F. Schlögl. Chemical reaction models for non-equilibrium phase transition. *Zeitschrift für Physik*, 253(2):147–161, 1972.

[14] T. Tian and K. Burrage. Stochastic models for regulatory networks of the genetic toggle switch. *Proc. Natl. Acad. Sci.*, 103(22):8372–8377, 2006.

[15] J. M. G. Vilar, H. Y. Kueh, N. Barkai, and S. Leibler. Mechanisms of noise-resistance in genetic oscillator. *Proc. Natl. Acad. Sci.*, 99(9):5988–5992, 2002.
